# *Amblyomma americanum* serpin 27 (AAS27) is a tick salivary anti-inflammatory protein secreted into the host during feeding

**DOI:** 10.1371/journal.pntd.0007660

**Published:** 2019-08-26

**Authors:** Lucas Tirloni, Tae Kwon Kim, Markus Berger, Carlos Termignoni, Itabajara da Silva Vaz, Albert Mulenga

**Affiliations:** 1 Department of Veterinary Pathobiology, College of Veterinary Medicine, Texas A&M University, College Station, United States of America; 2 Centro de Biotecnologia, Universidade Federal do Rio Grande do Sul, Porto Alegre, RS, Brazil; 3 Centro de Pesquisa Experimental, Hospital de Clínicas de Porto Alegre, Porto Alegre, RS, Brazil; 4 Departamento de Bioquímica, Universidade Federal do Rio Grande do Sul, Porto Alegre, RS, Brazil; 5 Faculdade de Veterinária, Universidade Federal do Rio Grande do Sul, Porto Alegre, RS, Brazil; Medical College of Wisconsin, UNITED STATES

## Abstract

Ticks successfully feed and transmit pathogens by injecting pharmacological compounds in saliva to thwart host defenses. We have previously used LC-MS/MS to identify proteins that are present in saliva of unfed *Amblyomma americanum* ticks that were exposed to different hosts. Here we show that *A*. *americanum* serine protease inhibitor (serpin) 27 (AAS27) is an immunogenic saliva protein that is injected into the host within the first day of tick feeding and is an anti-inflammatory protein that might act by blocking plasmin and trypsin functions. Although AAS27 is injected into the host throughout tick feeding, qRT-PCR and western blotting analyses indicate that the respective transcript and protein are present in high amounts within the first 24 h of tick feeding. Biochemical screening of *Pichia pastoris*-expressed recombinant (r) AAS27 against mammalian proteases related to host defense shows it is an inhibitor of trypsin and plasmin, with stoichiometry of inhibition indices of 3.5 and 3.8, respectively. Consistent with typical inhibitory serpins, rAAS27 formed heat- and SDS-stable irreversible complexes with both proteases. We further demonstrate that rAAS27 inhibits trypsin with *k*_*a*_ of 6.46 ± 1.24 x 10^4^ M^-1^ s^-1^, comparable to serpins of other tick species. We show that native AAS27 is part of the repertoire of proteins responsible for the inhibitory activity against trypsin in crude tick saliva. AAS27 is likely utilized by the tick to evade the hosts inflammation defense since rAAS27 blocks both formalin and compound 48/80-induced inflammation in rats. Tick immune sera of rabbits that had acquired resistance against tick feeding following repeated infestations with *A*. *americanum* or *Ixodes scapularis* ticks reacts with rAAS27. Of significant interest, antibody to rAAS27 blocks this serpin inhibitory functions. Taken together, we conclude that AAS27 is an anti-inflammatory protein secreted into the host during feeding and may represent a potential candidate for development of an anti-tick vaccine.

## Author summary

Ticks are blood-feeding arthropods that salivate while they puncture host skin in their search of blood. Tick saliva contains hundreds of compounds that have anti-coagulant, vasodilatory, anti-inflammatory, and immunomodulatory functions. While helping the vector to feed, tick saliva also modifies the site where pathogens are injected and in many cases facilitates the infection process. For this reason, tick salivary proteins can be targets to control tick and tick-borne diseases. Serpins are thought to control the tick’s evasion of the host’s serine protease-mediated defense pathways such as inflammation and blood coagulation. In this study, we report that *Amblyomma americanum* ticks secrete an anti-inflammatory serpin into the host during feeding. This work emphasizes the importance of understanding the functional roles of tick saliva proteins to tick feeding physiology to identify new targets in development of novel strategies for tick and tick-borne diseases control and also to search and find new potentially pharmacological active compounds.

## Introduction

The lone star tick *Amblyomma americanum* is a hard tick species of medical and veterinary importance in the United States and Mexico [1–3]. This tick species is a known vector of a number of tick-borne diseases (TBD) agents including *Borrelia lonestari*, the causative agent of southern tick-associated rash illness (STARI) [4]; *Francisella tularensis*, the causative agent of tularemia in humans [5,6]; *Ehrlichia chaffeensis* and *Ehrlichia ewingii*, the causative agents of human ehrlichiosis [7,8]; and *Rickettsia amblyommii*, the causative agent of rickettsiosis of the spotted fever group [9,10]. A recent study demonstrated the vector capability of *A*. *americanum* in acquiring, maintaining and transmitting *Rickettssia rickettsii* isolates originating from two different geographical regions of the US [11]. Furthermore, the possible role of *A*. *americanum* to transmit Heartland and Bourbon viruses was documented [12,13]. Likewise, *A*. *americanum* is a competent vector of *Cytauxzoon felis* and *Theileria cervi* pathogens affecting domestic cats and white-tailed deer, respectively [2,14].

In absence of vaccines against major ticks and TBD agents, current tick control strategies rely mostly on the use of chemical acaricides, even though selection of resistant tick populations to most used acaricides has been confirmed [15,16]. This is recognized as a drawback to a successful tick control, and not to mention environment and food chain contamination hazards. Immunization of animals against tick feeding emerged as a sustainable tick control strategy [17,18]. In the effort to find effective targets for an anti-tick vaccine development, understanding tick-feeding physiology could lead to the discovery of important tick saliva proteins that can be targeted for anti-tick vaccine development.

Ticks accomplish blood meal feeding by disrupting host tissue and sucking up blood from the feeding lesion. This feeding style triggers host defense responses including pain, hemostasis, inflammation, complement activation, and tissue repair responses [19]. Serine proteases mediate some of the host defense pathways to tick feeding and are controlled in some pathways by inhibitors belonging to the serine protease inhibitor (serpin) family [20]. From this perspective, ticks were thought to inject serpins into the host to mediate evasion of host defenses. The presence of serpins in tick saliva was well demonstrated though saliva proteomic studies [21–23] and recent evidence shows that some of the tick-encoded serpins are functional inhibitors of host defense system proteases [24,25]. We have also recently described *A*. *americanum* serpins that are expressed by both male and female *A*. *americanum* ticks [26]. In a recent study, we used LC-MS/MS to identify proteins in saliva of unfed *A*. *americanum* that were stimulated to start feeding on different hosts (rabbits, dogs and humans) [27]. The rationale to focus on early stages of tick feeding is that most TBD pathogens are transmitted within the first 72 h of tick feeding [28], thus identifying tick saliva proteins that are important for initial tick feeding success could serve as ideal antigens targets in designing effective anti-tick vaccines against tick feeding and pathogen transmission. However a major limitation to discovery of effective anti-tick vaccines is the lack of understanding the functional roles of these molecular components in tick feeding. To begin understanding the functional roles to tick saliva serpins in tick feeding success, this study was undertaken to characterize *A*. *americanum* serpin 27 (AAS27), one of the serpins that is highly abundant in saliva of unfed adult female ticks that were stimulated to start feeding on different hosts (rabbit, dog, and human) [27]. Our data show that, native AAS27 is likely one of the tick saliva proteins that mediate the tick’s evasion of the host’s inflammatory defense to tick feeding.

## Results

### AAS27 is expressed in all life stages and multiple tick organs

**Fig 1:**
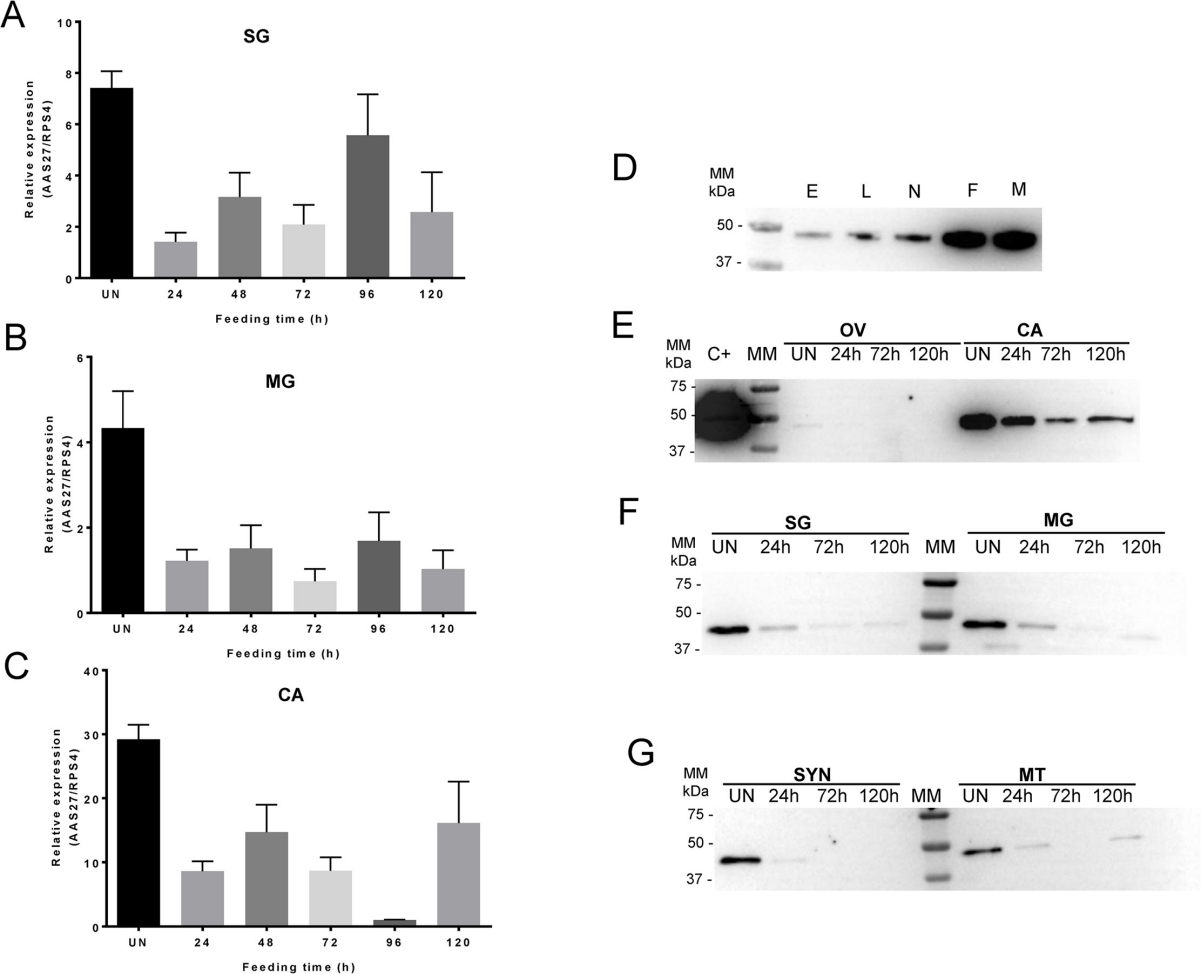
AAS27 is highly transcribed at adult unfed stage. (A-C) AAS27 relative expression analysis in unfed (UN), 24, 48, 72, 96, and 120 h fed adult female tick dissected tissues: salivary glands (SG), midguts (MG) and remnants as carcass (CA) analyzed by qRT-PCR. In three biological replicates, relative expression levels of AAS27 were analyzed using the 2^−ΔΔCt^ method using ribosomal protein S4 (RPS4) transcript as internal reference. The lowest expressed time point was used as a calibrator for each tissue. (D-G) AAS27 qualitative expression analysis were performed by western blot in in unfed (UN), 24, 72, and 120h fed tick dissected salivary glands (SG), midguts (MG), synganglion (SYN), Malpighian tubules (MT), ovary (OV) and remnants as carcass (CA), eggs 22 days after oviposition (E), unfed larvae (L), unfed nymphs (N), unfed adult females (F) and males (M). Protein extracts were subjected to western blotting analyses using a monospecific antibody (purified) against yeast-expressed rAAS27 (0.30 μg/μL in a 1:500 dilution). rAASrAAS-27 (500 ng) was used as positive control (C+).

A spatial and temporal transcription profile of AAS27 was used to determine its relationship to the tick-feeding process (Fig 1A–1C). Results from qRT-PCR analysis show that AAS27 mRNA is transcribed in salivary glands (SG), midguts (MG), and carcass (CA, tissue remnant after removal of SG and MG) (Fig 1A–1C). In all tissues analyzed transcript abundance was substantially higher at the unfed stage, and being reduced within 24 h of attachment. With reference to the 24 h feeding time point, there is an apparent transcript increase in SG at 96 h (Fig 1A), and at 48 and 120 h in CA (Fig 1C).

Fig 1D–1G summarizes native AAS27 protein expression in different life stages and tick organs of unfed and fed adult female *A*. *americanum*. Native AAS27 is expressed in eggs at 22 days after eggs were laid, unfed larvae, nymphs, female and male adults ticks (Fig 1D). Consistent with qRT-PCR data, native AAS27 protein is apparently highly abundant at the unfed stage in all analyszed tick organs: salivary glands (SG), midgut (MG), Malpighian tubules (MT), synganglion (SYN), ovary (OV), carcass (CA) (Fig 1E–1G). The mature AAS27 is 377 amino acids long with a 41 kDa calculated molecular mass [26]. Immunoblot analysis detected a single native AAS27 protein band within the expected size range of between 37 and 50 kDa. Pre-immune sera did not show binding (S1 Fig) demonstrating specificity of the reaction. Please also note in S1 Fig, we have provided images of full gels that have been cropped and presented in Fig 1D–1G.

Given the fact that AAS27 protein is highly abundant in unfed ticks, we decided against conducting RNAi silencing of this protein to determine its significance in tick feeding. Based our previous work [29,30], we reasoned that RNAi disruption of encoding mRNA might not sufficiently deplete AAS27 protein that was translated prior to RNAi silencing, and thus our results might not be informative. Our preliminary attempts to deplete mRNA from unfed ticks as described [29,30] before conducting RNAi silencing analyses were unsuccessful, and we did not proceed with RNAi silencing.

### AAS27 is expressed as a glycoprotein in *Pichia pastoris*

AAS27 was successfully expressed using *Pichia pastoris* system (S2 Fig). Approximately 17 mg of purified rAAS27 was obtained from culture supernatant (1 L). Purified rAAS27 migrates as a 50 kDa band on 12% SDS-PAGE under reducing conditions (S2 Fig). When treated with glycosidases we could observe a downward molecular weight shift suggesting rAAS27 is expressed as a glycoprotein (S2 Fig).

### AAS27 has basic patches but does not bind glycosaminoglycans (GAGs)

Comparative modeling using neuroserpin tertiary structure as a template showed that AAS27 predicted tertiary structure retains the typical serpin fold (S3 Fig). Surface electrostatic potential was calculated and showed the presence of putative basic patches on AAS27 model (dashed circles in S3 Fig). There is evidence that basic patches on serpins could bind GAGs [31]. We used two different strategies to check if rAAS27 is able to interact with two classes of GAGs (heparin/heparan sulfate and chondroitin/dermatan sulfate): (i) an affinity purification using heparin-Sepharose (S4 Fig) and (ii) GAG-binding plate assay (S4 Fig). These results show that putative GAG-binding sites predicted on AAS27 model were not able to bind GAGs used in this study. S4 Fig shows that rAAS27 did not bind onto heparin, since rAAS27 was detected only in run-through (lane 2) and in fractions eluted with washing buffer (lane 3). Similarly, no interaction of rAAS27 with heparin, heparan sulfate, dermatan sulfate, and chondroitin sulfate was observed using a GAG-binding plate assay (S4 Fig).

### AAS27 is an inhibitor of trypsin and trypsin-like serine proteases

**Fig 2:**
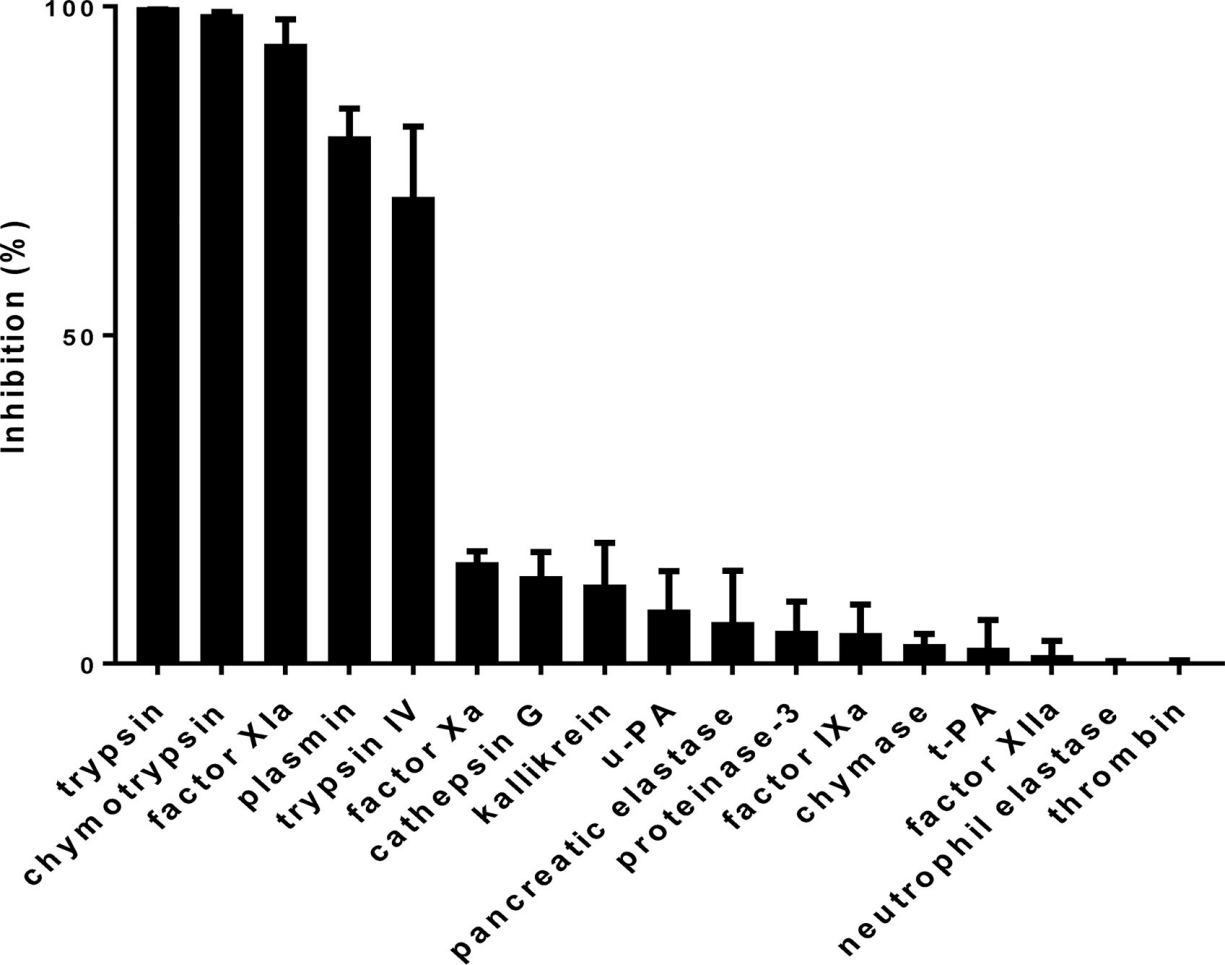
rAAS27 inhibits trypsin-like serine proteases. rAAS27 (1 μM) was tested against 17 different host-derived serine proteases, including: rat trypin IV (20 nM), pancreatic bovine α-chymotrypsin (1.4 nM), pancreatic porcine elastase (61.8 nM), human proteinase-3 (280 nM), human chymase (21.7 nM), pancreatic bovine trypsin (0.3 nM), pancreatic porcine kallikrein (10 nM), human thrombin (19.2 nM), human neutrophil cathepsin G (425.5 nM), human plasmin (5.3 nM), human factor XIa (3.7 nM), bovine factor IXa (314.4 nM), human factor XIIa (15 nM), human neutrophil elastase (14.9 nM), human t-PA (23.6 nM), human u-PA (29.6 nM), and human factor Xa (2.3 nM). rAAS27 and protease were pre-incubated for 15 minutes at 37°C in 20 mM Tris-HCl, 150 mM NaCl, BSA 0.1%, pH 7.4 buffer. The corresponding substrate for each enzyme was added in a 100 μL final reaction volume and substrate hydrolysis was measured at OD_405nm_ every 11s for 15 min at 30°C. The percent enzyme activity inhibition level was determined as described in Material and Methods section. Data are presented as mean ± standard deviation of three independent replicate readings.

**Fig 3:**
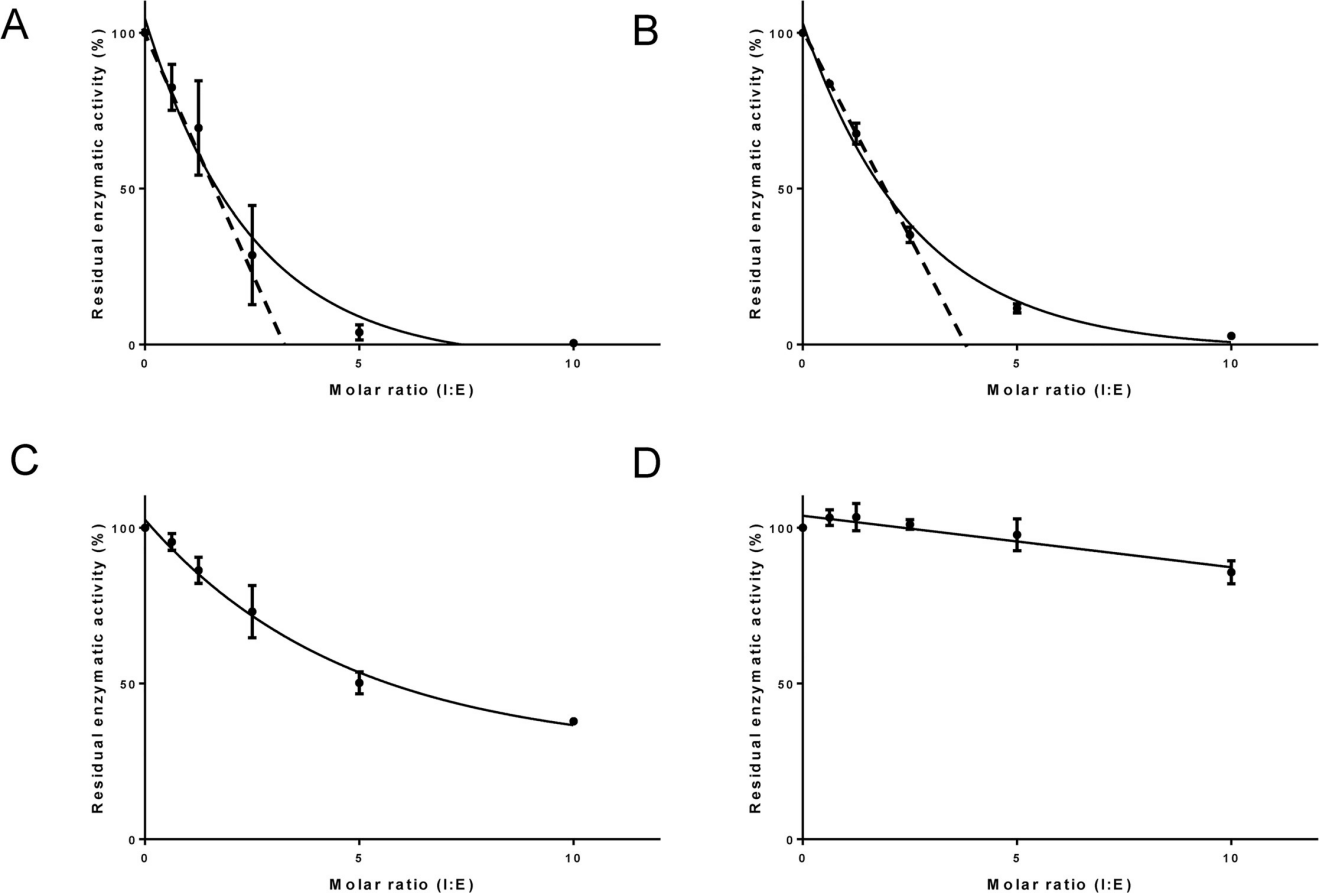
rAAS27 is an effective inhibitor of trypsin and plasmin. Residual protease activity in the presence and absence of rAAS27 was evaluated by pre-incubating rAAS27 for 1 h at 37°C with (A) trypsin (2 nM), (B) plasmin (34 nM), (C) chymotrypsin (2.8 nM), and (D) factor XIa (3.7 nM), resulting in molar ratios (serpin:protease) ranging from 0 to 10. Protease activity was measured using specific colorimetric substrate for each protease as described in Materials and Methods. The data were plotted as residual protease activity (V_i_/V_0_) versus molar ratio (serpin:protease). The SI was determined by linear regression to the initial points of inhibitory curve. Data are presented as mean ± standard deviation of three independent replicate readings.

**Fig 4:**
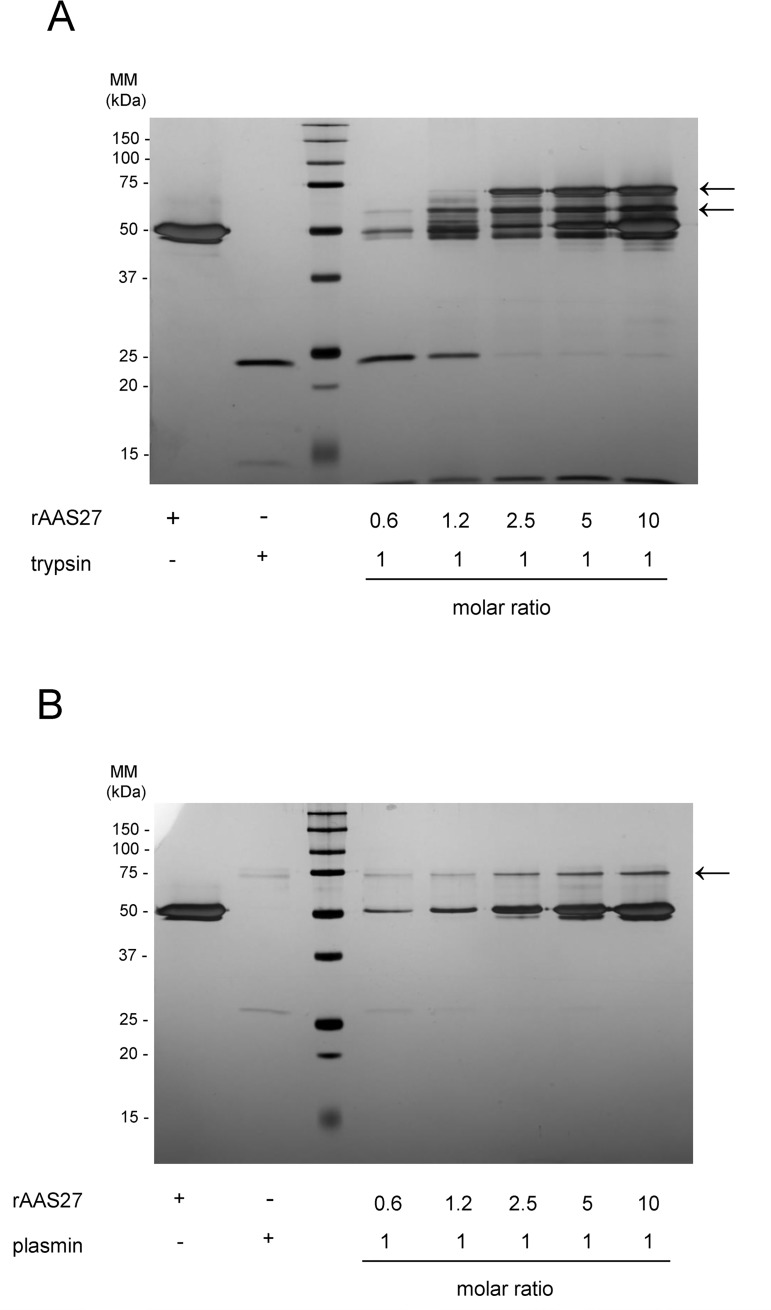
rAAS27 forms heat and SDS-stable complex with trypsin and plasmin. Increasing amounts of rAAS27 were pre-incubated for 1 h at 37°C with a constant concentration of (A) trypsin (0.1 μg) and (B) plasmin (0.2 μg), resulting in molar ratios varying from 0.6:1 to 10:1 (serpin:protease). Samples were resolved on 12% SDS–PAGE and silver-stained to identify SDS-stable complexes (indicated by arrows).

**Fig 5:**
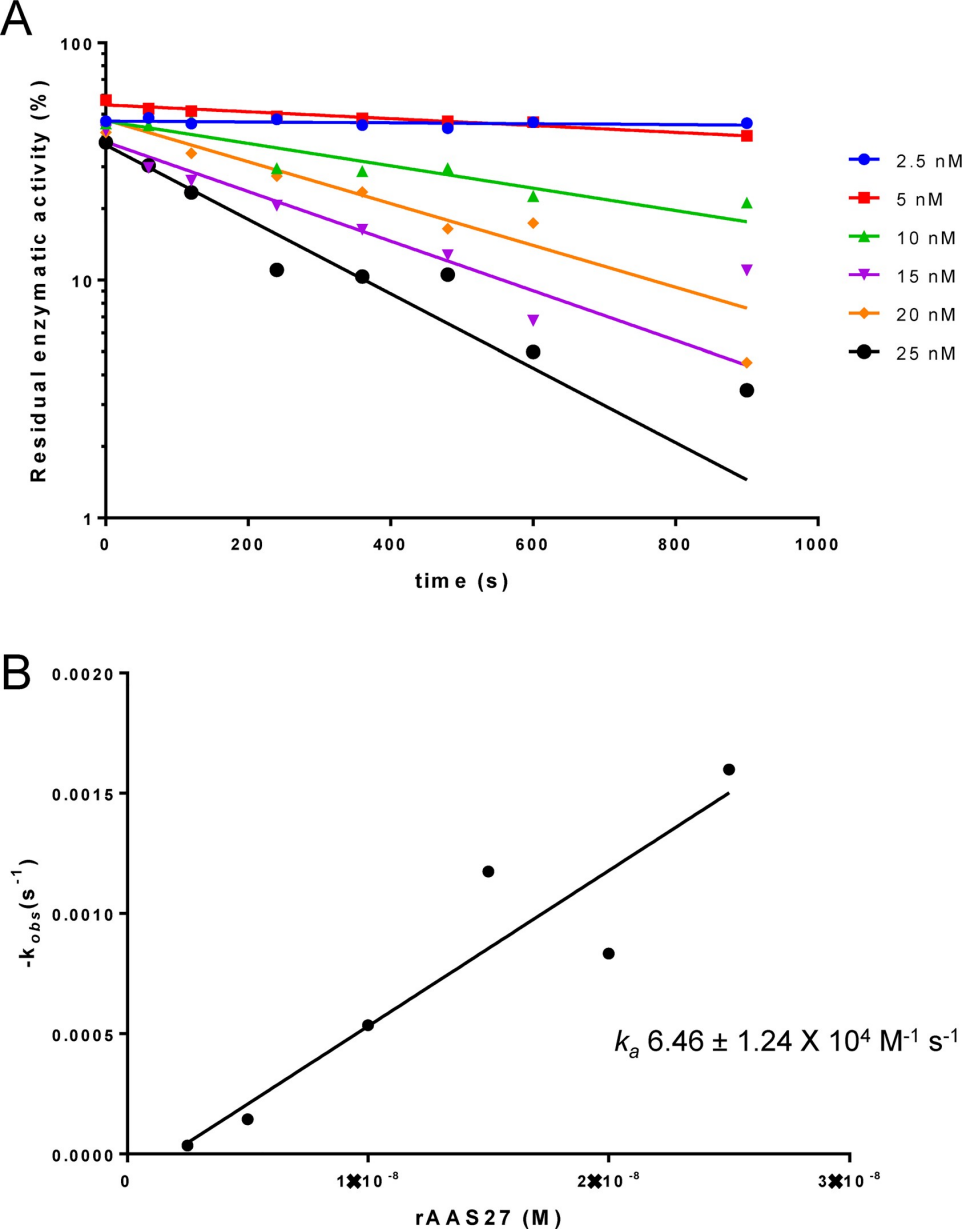
rAAS27 is an effective inhibitor of trypsin. Discontinuous assay of the inhibition of trypsin by rAAS27. (A) Semilogarithmic plots of residual enzymatic activity of trypsin versus time of incubation for reactions at various concentrations of rAAS27 (2.5–25 nM). (B) Plot of *k*_*obs*_ as a function of rAAS27 concentration. Linear regression of the slope represents the second-order rate constant *k*_*a*_ for the inhibition of trypsin by rAAS27.

The inhibitory profile of rAAS27 was tested against 17 mammalian serine proteases related to host defense pathways as previously described [32,33]. Incubation of rAAS27 with each protease in a molar excess showed that rAAS27 (1 μM) inhibits the activity of bovine pancreatic trypsin (0.3 nM) by 99%, the activity of pancreatic bovine α-chymotrypsin (1.4 nM) by 98%, the activity of human plasmin (5.3 nM) by 94%, the activity of human factor XIa (3.7 nM) by 80%, and the activity of rat trypsin IV (20 nM) by 71% (Fig 2). To check the inhibitory efficiency, stoichiometry of inhibition (SI) index were calculated. Accordingly, the SI index for rAAS27 was 3.5 against bovine pancreatic trypsin (Fig 3A), 3.8 against human plasmin (Fig 3B), and higher than 10 against chymotrypsin (Fig 3C), human factor XIa (Fig 3D). The rAAS27 mechanism of action as a typical inhibitory serpin was confirmed by the inability of heat and SDS to dissociate it from bovine pancreatic trypsin (Fig 4A) and human plasmin (Fig 4B). After incubation of trypsin or plasmin with rAAS27, high molecular weight complexes were formed (as indicated by arrows in Fig 4) and there was an apparent increase in consumption of protease with higher concentrations of rAAS27. The formation of a covalent complex was observed on 12% SDS-PAGE (Fig 4) as a band migrating at approximately the same position as the sum of the target protease and the cleaved rAAS27 (approximately 75 kDa). These irreversible complexes between rAAS27 and the target proteases were observed at similar molar ratios comparable to SI assays (Fig 3A and Fig 3B). The association rate constant (*k*_*a*_) of rAAS27 with pancreatic bovine trypsin was measured under pseudo-first order conditions using a discontinuous assay [34]. The *k*_*a*_ for the interaction of rAAS27 and pancreatic bovine trypsin was determined as *k*_*a*_ 6.46 ± 1.24 x 10^4^ M^-1^ s^-1^, demonstrating rAAS27 is a fast and effective inhibitor of trypsin (Fig 5).

### Native AAS27 secreted in tick saliva has anti-trypsin activity

**Fig 6:**
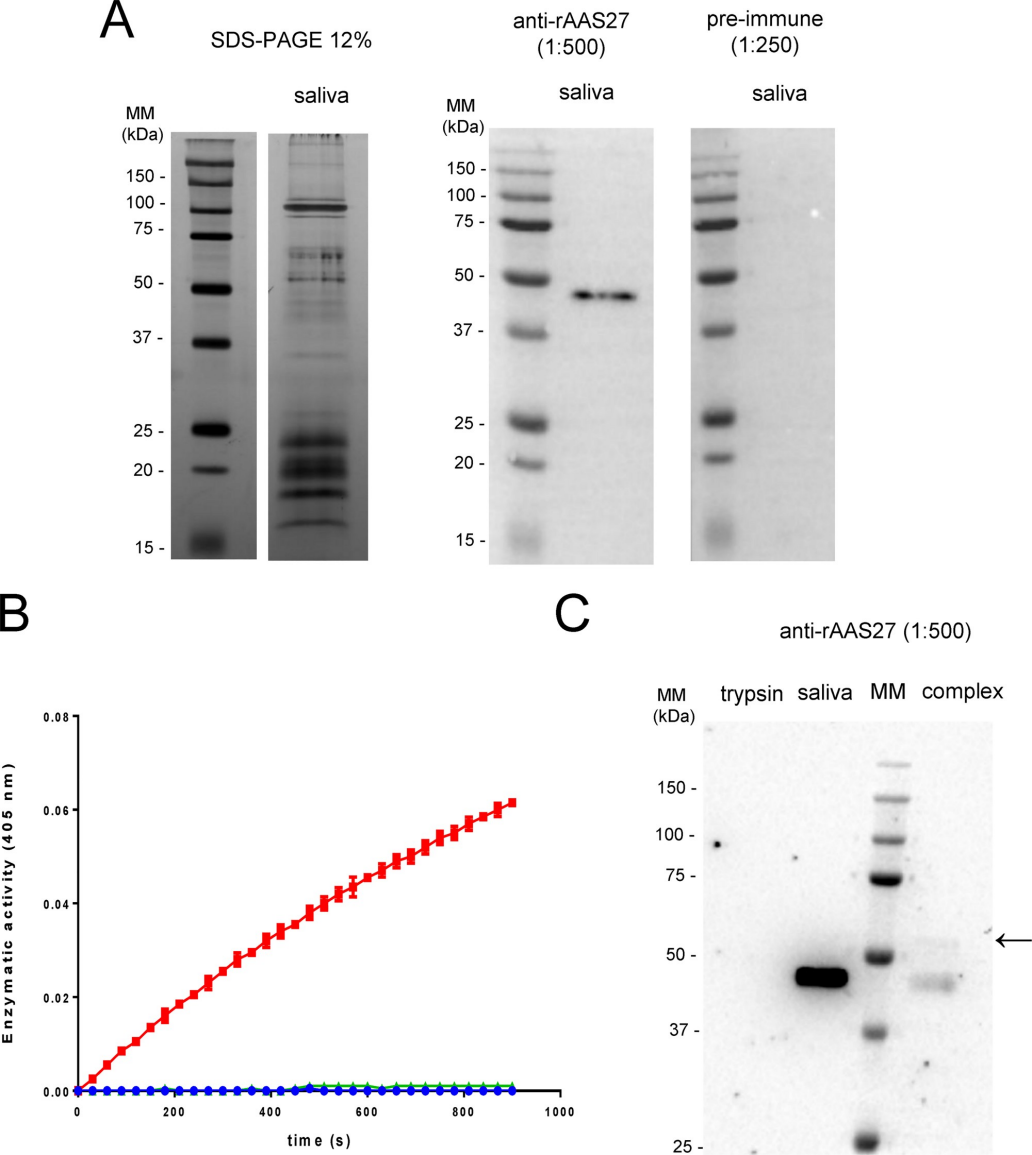
Anti-trypsin activity and native AAS27 were observed in *A*. *americanum* tick saliva. Tick saliva was collected from *Amblyomma americanum* unfed adult females as described in Material and Methods. (A) Tick saliva harvested from unfed ticks was resolved on 12% SDS-PAGE gels and subjected to silver staining (2 μg of total protein resolved) and western blot analysis (1 μg total protein resolved) using a monospecific antibody produced against yeast-expressed rAAS27 (1.0 μg/μL in a 1:500 dilution) and purified total IgG from pre-immune serum (0.50 μg/μL in a 1:250 dilution). (B) Tick saliva (1 μg) was tested against pancreatic bovine trypsin (2 nM). Tick saliva and protease were pre-incubated for 15 min at 37°C in 20 mM Tris-HCl, 150 mM NaCl, Tween 0.01%, pH 7.4 buffer. Substrate was added in a 100 μL final reaction volume and substrate hydrolysis was measured at OD_405nm_ every 15s for 15 min at 30°C. Data presented is representative of three independent replicates and results are plotted as mean of a reading in duplicate. (C) Tick saliva harvested from ticks (1 μg) was incubated with trypsin (0.1 μg) for 1 hour at 37°C. Samples were subjected to SDS-PAGE 12% followed by western blot analysis using anti-rAAS27 monospecific antibody (1.0 μg/μL in a 1:250 dilution).

Given the fact that native AAS27 is present in tick saliva, we investigated anti-trypsin inhibitory activity in whole tick saliva. To prevent any contamination from host proteins, whole tick saliva was collected from unfed adult *A*. *americanum* female ticks (Fig 6A). In Fig 6B we used the monospecific antibody to rAAS27 to confirm that native AAS27 was present in whole tick saliva as demonstrated by specific reactivity of the expected ~41 kDa protein band. Since we could identify native AAS27 in *A*. *americanum* tick saliva, we next investigated the effect of saliva on trypsin amidolytic activities. We show that tick saliva (1 μg total protein) completely inhibited the activity of trypsin (2 nM) (Fig 6C). When tick saliva was co-incubated with trypsin, we could observe a covalent complex formation between native AAS27 and the protease (Fig 6D). Altogether, these results showed that native AAS27 is present in tick saliva and has anti-trypsin activity.

### AAS27 is among tick salivary immunogens that provoke anti-tick immunity in rabbits that are repeatedly infested with *A*. *americanum* or *Ixodes scapularis* ticks

**Fig 7:**
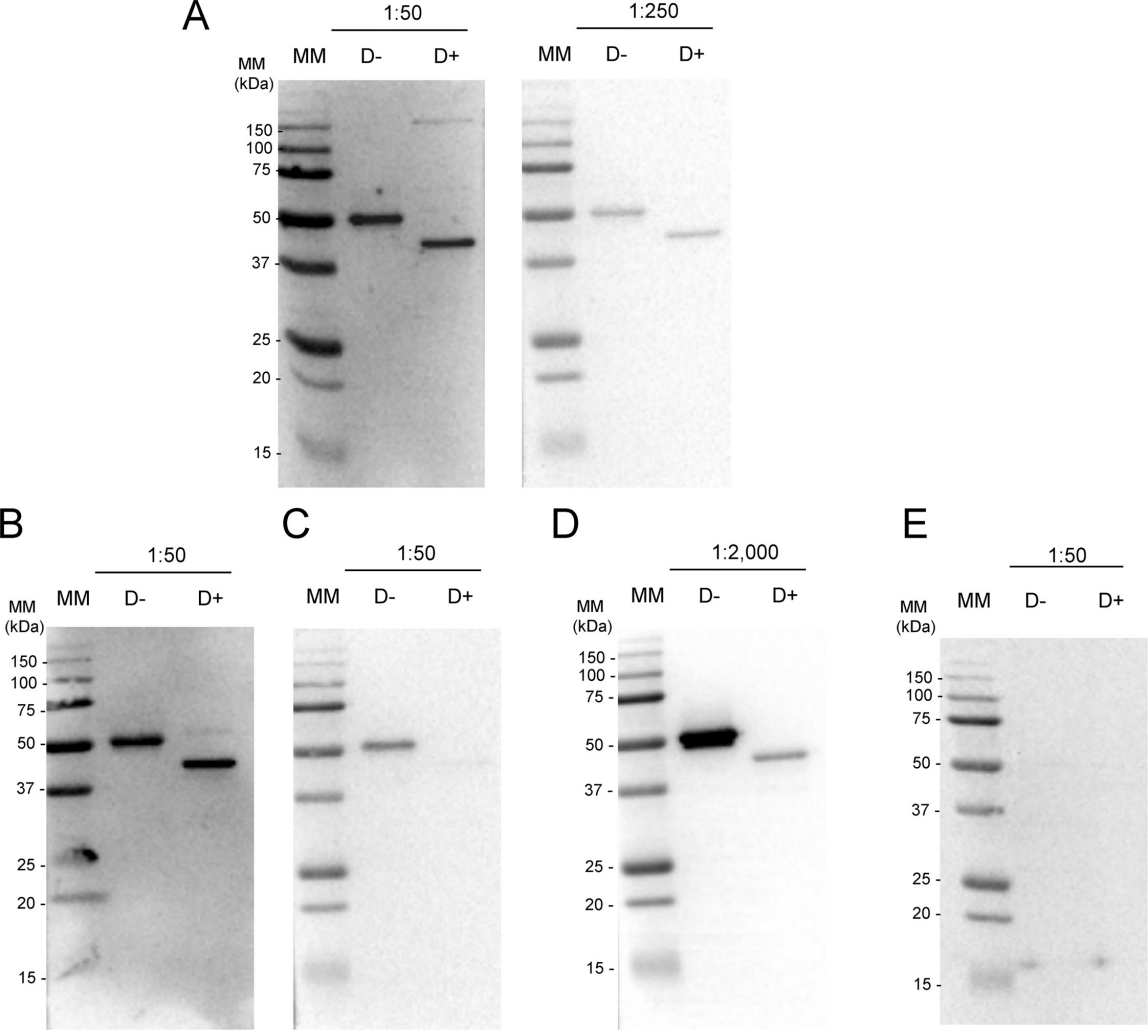
rAAS27 binds antibodies to both *Amblyomma americanum* and *Ixodes scapularis* tick saliva proteins. Purified rAAS27 was treated with deglycosylation enzyme mix (D+) or without treatment (D-) and resolved on a 12% SDS-PAGE following western blotting analysis with: (A) rabbit serum generated by repeated *A*. *americanum* adult tick infestation (dilution 1:50 and 1:250), (B) rabbit serum generated by repeated adult *I*. *scapularis* infestations (dilution 1:50), (C) rabbit serum generated by nymph *I*. *scapularis* infestations (dilution 1:50), (D) monospecific antibody produced against yeast-expressed rAAS27 was used as positive control (0.30 μg/μL in a 1:2,000 dilution) and (E) and rabbit pre-immune serum (dilution 1:50).

Repeated tick infestation of animals can induce protective host immunity that reduces tick feeding efficiency [35]. In order to determine if native AAS27 was among immunogens that provoke a humoral immune response in rabbits that were repeatedly infested with *A*. *americanum*, rAAS27 (glycosylated and de-glycosylated) was subjected to western blotting analysis using rabbit antibodies generated during experimental infestation with *A*. *americanum* and *I*. *scapularis* ticks (Fig 7). Similar to binding pattern of the monospecific antibody to rAAS27 (positive control, Fig 7A), serum antibodies from rabbits repeatedly infested with adult *A*. *americanum* (Fig 7B) and *I*. *scapularis* (Fig 7C) as well as nymph *I*. *scapularis* ticks (Fig 7D) bound to rAAS27, but not to pre-immune sera (Fig 7E). It is notable that rabbit antibodies to *I*. *scapularis* adult tick saliva proteins weakly bound de-glycosylated rAAS27 (Fig 7C). Taken together, this data demonstrates that rAAS27 is one of the multiple immunogens that collectively provokes protective humoral immune response against tick feeding in rabbits that are repeatedly infested by *A*. *americanum* or *I*. *scapularis* ticks.

### Antibody to rAAS27 blocks its inhibitory functions against trypsin

**Fig 8:**
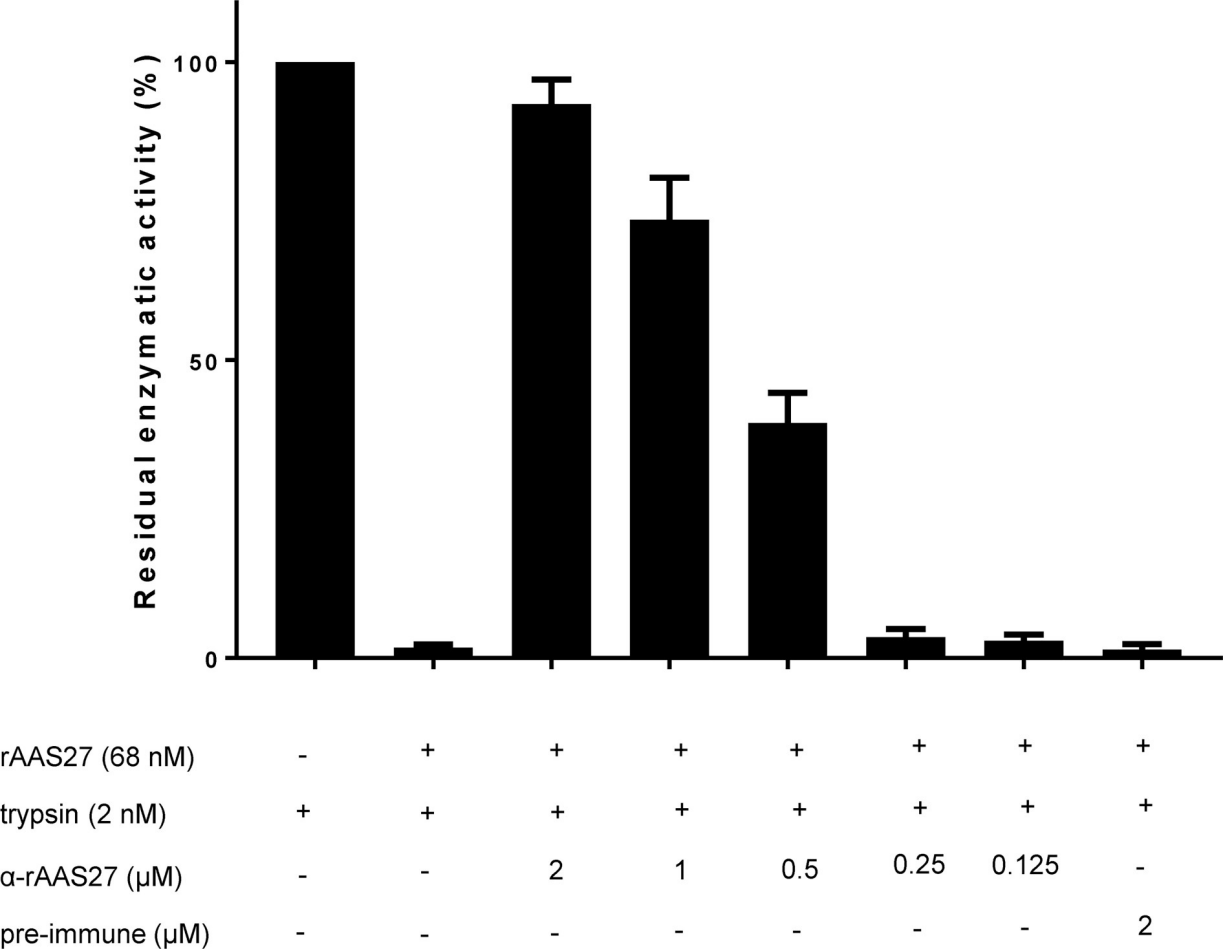
Monospecific antibody to rAAS27 dose responsively inhibits its inhibitory activity. Purified IgG (various doses) from monospecific serum anti-rAAS27 or pre-immune (PI) serum were incubated with rAAS27 (68 nM at 37°C for 30 min before the addition of trypsin (1.5 nM) following an additional 15-min incubation at 37°C. Subsequently, the trypsin substrate was added and protease kinetics was monitored for 15 min every 15 s. Data is reported as residual enzymatic activity.

Given the fact that rAAS27 was immunogenic, we were curious to investigate if antibodies to rAAS27 affected its function. Of significant interest, monospecific antibodies to rAAS27 significantly neutralized the inhibitory function of rAAS27 against trypsin in a dose dependent manner (Fig 8). Purified IgG from pre-immune serum was used as control and did not affect serpin inhibitory activity (Fig 8).

### AAS27 possesses anti-inflammatory properties *in vivo*

**Fig 9:**
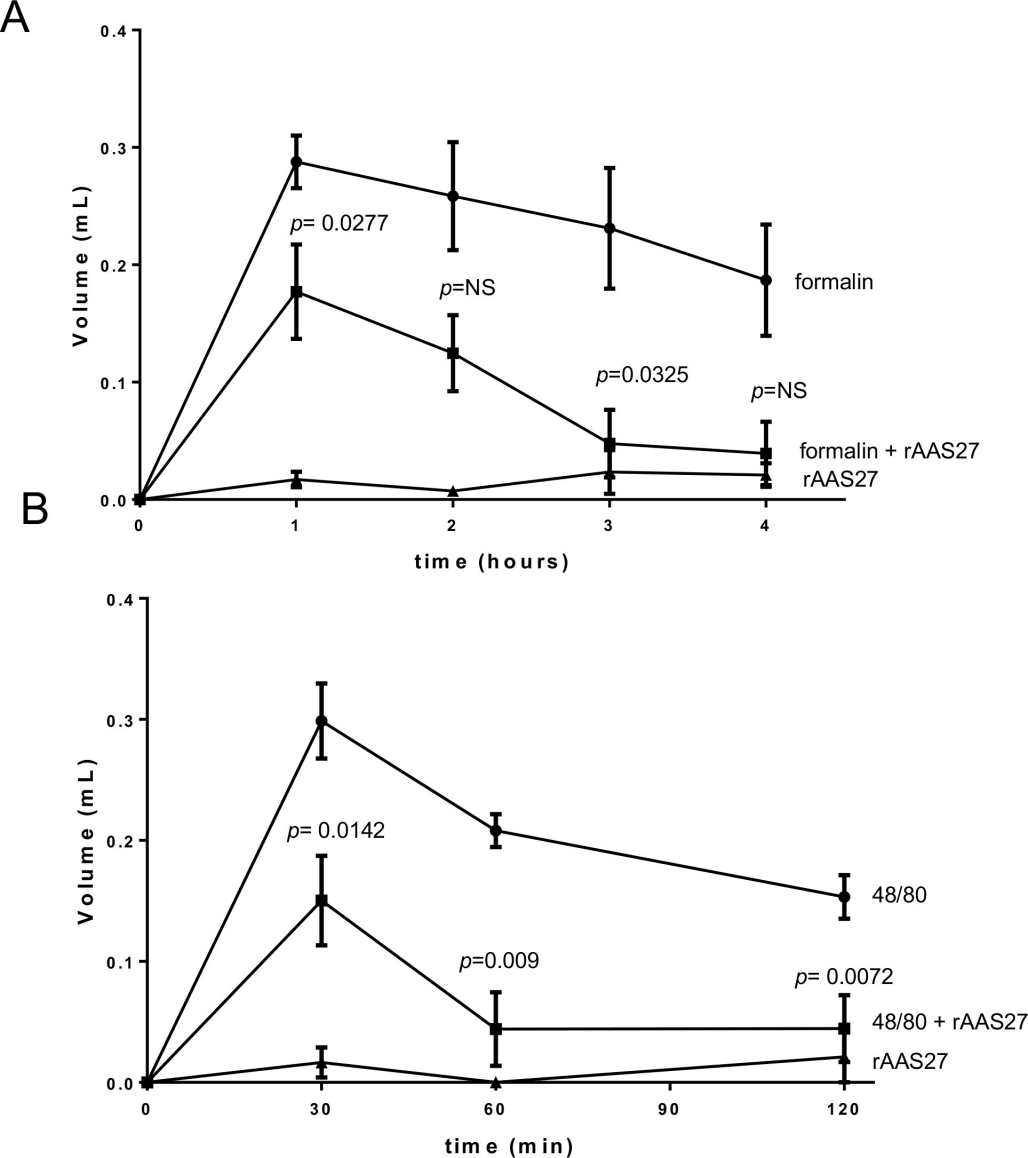
rAAS27 blocks formalin- and compound 48/80-induced acute inflammation *in vivo*. (A) Rat paw edema induced by intradermal injection of 100 *μ*L of formalin (0.03%) in saline (circles), or co-injected with 25 *μ*g of rAAS27 (squares), or 100 *μ*L of rAAS27 only (triangles). (B) Rat paw edema induced by intradermal injection of 100 *μ*L of compound 48/80 (1 *μ*g) in saline (circles), or co-injected with 25 *μ*g of rAAS27 (squares), or 100 *μ*L of rAAS27 only (triangles). Edema formation was estimated using a digital pletismometer before injection of the agonist at different intervals. Posterior paws from 5 animals were used for each data point. Unpaired t-test was used for statics analysis and *p* ≤0.05 was considered statistically significant.

The finding that rAAS27 inhibits trypsin and plasmin suggests this serpin might attenuate inflammation and could contribute to the immunomodulatory activity of tick saliva [36–38]. We evaluated the effects of rAAS27 in a rat model of acute inflammation induced by formalin and compound 48/80 (an agonist of mast cell degranulation). Injecting compound 48/80 into the skin degranulates mast cells as occurs during to tick-feeding [39–41]. With respect to normal physiological functions, mast cells are known to regulate vasodilation, vascular homeostasis, innate and adaptive immune responses, and angiogenesis [42]. The cytoplasm of the mast cell contains large granules that store inflammatory mediators, including histamine, heparin, a variety of cytokines, chondroitin sulfate, and neutral proteases, including chymase and tryptase [43]. In animal models, injection of compound 48/80 induces mast cell degranulation accompanied by thermal hyperalgesia, tissue edema, and neutrophil influx [44]. Similarly, injection of formalin into mouse paw releases locally several forms of active trypsin-like serine proteases. These proteases generate PAR-derived peptides and activates cells via PAR-2-dependent mechanism, resulting in an acute inflammatory response characterized by edema formation in the paw [36]. When inoculated into the rat footpad, formalin and compound 48/80 induces an acute inflammatory process characterized by edema formation. This effect was confirmed by the increase in paw thickness reaching a maximum 1 h post injection for formalin (Fig 9A) and 30 min post injection for compound 48/80 (Fig 9B). In the presence of rAAS27 (25 μg/paw) edema formation was significantly reduced. More specifically, in the formalin-induced paw edema assay the decrease in edema formation reached 38% (*p* = 0.027) at 1 hour and 82% (*p* = 0.027) at 3 hours post-treatments, compared to positive control (formalin-injected footpads). The inhibition observed after 2 and 4 hours of formalin injection with rAAS27 was 51% and 78%, compared with positive control, but it was not statistically significant (Fig 9A). Similarly, in the compound 48/80-induced paw edema assay, the decrease in edema formation reached 49% (*p* = 0.014) at 30 min, 78% (*p* = 0.009) at 60 min, and 71% (*p* = 0.007) at 120 min post-injections (Fig 9B).

**Fig 10:**
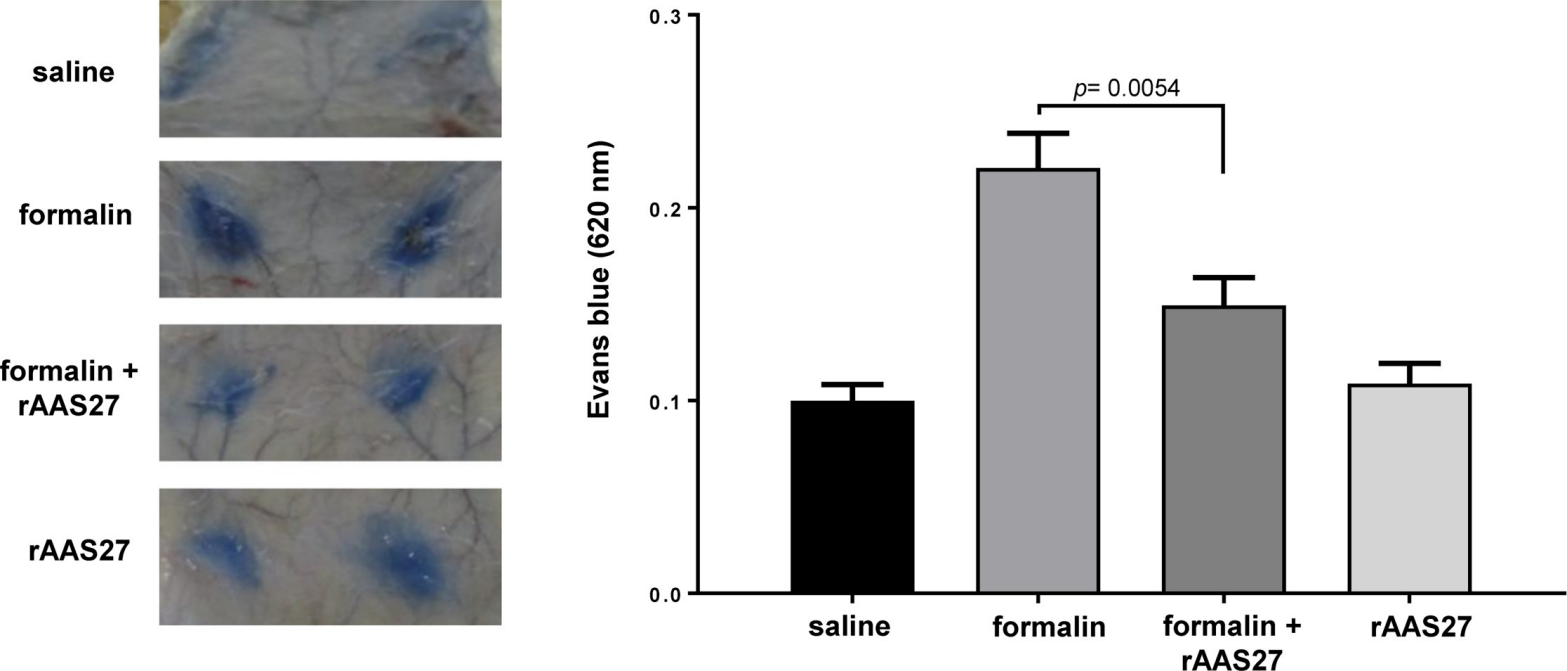
rAAS27 reduces formalin-induced vascular permeability *in vivo*. Vascular permeability (Miles assay) was performed by intravenous injection of 700 *μ*L Evan’s blue dye into the tails of rats. Ten minutes later, 100 *μ*L of (i) saline, (ii) formalin 0.3% only, (iii) formalin 0.3% co-injected with rAAS27 (25 *μ*g), or (iv) only 25°*μ*g rAAS27 was injected intradermally on the back of anhestized animals. Sixty minutes after injecting the Evans blue dye, animals were sacrificed and skin removed and spots at injection sites were photographed. The extravasated Evans blue dye was estimated after extraction with formamide and reading at 620 nm. Results are the average of experiments obtained with 6 animals (02 spots per animal). *P = 0.0054 (t-test). Unpaired t-test was used for statics analysis and *p* ≤0.05 was considered statistically significant.

Intradermically injected formalin increases vascular permeability into the subcutaneous tissue and can be estimated using the Miles assay by measuring Evans blue dye fluid extravasation in rat skin [45]. Fig 10 shows that formalin significantly increased the vascular permeability into skin subcutaneous tissue (Fig 10A), while rAAS27 (25 μg/spot) prevented this stimulatory response almost completely (*p* = 0.005) (Fig 10B).

## Discussion

Ticks are blood feeding arthropods that salivate while they puncture host skin in their search for blood. Tick saliva contains hundreds of compounds that have anti-coagulant, vasodilatory, anti-inflammatory, and immunomodulatory functions [19,21,22]. Inflammatory host response induced by tick feeding is expected to create a hostile environment for foreign pathogens, but is alleviated by tick saliva [46]. The anti-inflammatory properties of tick saliva is already well-documented [47,48], however the molecular identity of main anti-inflammatory molecules in *A*. *americanum* saliva is not fully known. In a previous study, we identified by LC-MS/MS over 300 tick saliva proteins that are present in unfed *A*. *americanum* ticks that were stimulated to feed on different hosts, of which AAS27 was the most abundant serpin identified in all treatments [27]. In this study, we demonstrate the functional characterization of AAS27 [26], as an anti-inflammatory serpin that *A*. *americanum* ticks inject into the host during feeding.

Our spatial-temporal expression analysis showed that both mRNA transcript and native protein of AAS27 is expressed in salivary glands, midgut, Malpighian tubes, synganglion, and carcass (the remnants after removal of other organs). Of significance, both transcript and proteins of AAS27 are highly abundant at the unfed stage, suggesting this serpin is secreted into the host within 24 h of the tick starting to feed. The detection of AAS27 in internal organs suggest this serpin may also regulate other endogenous biological systems [25].

In addition to confirming that native AAS27 is present in tick saliva, consistent with our previous proteomic study [27], here we show that antibodies raised against rAAS27 are able to recognize the native serpin that is present in tick saliva (Fig 6). Additionally, immune serum from rabbits that were repeatedly infested with *A*. *americanum* ticks recognize rAAS27, demonstrating this protein is injected into hosts during tick feeding and is potentially one of the multiple immunogens that collectively provoke protective immunity against tick feeding in rabbits that are repeatedly infested by *A*. *americanum* ticks (Fig 7). Data showing cross-reactivity among sera from hosts infested with different tick species suggest that AAS27 homologs may be present in saliva of other ticks species, highlighting the potential use of salivary serpins in a universal anti-tick vaccine [49] to interfere with normal tick feeding and against subsequent pathogen transmission.

Consistent with previous findings that some serpins are glycoproteins [50], this study shows that rAAS27 is expressed in *Pichia pastoris* as a glycoprotein (S2 Fig). Tertiary serpin structure typically contains three β-sheets, eight or nine α-helices, and a reactive center loop (RCL) [50]. The RCL is a solvent exposed flexible stretch of 21 amino acid residues positioned between β-sheets sA and sC and acts as a bait for its cognate protease [50]. Comparative modeling using human neuroserpin as a template showed that the rAAS27 predicted tertiary structure retains a typical serpin fold (S3 Fig). The RCL sequences displays hypervariability due to lack of structural constraints, and the P_1_ residue in the RCL is critical to define the specificity of a serpin for a particular protease. The predicted P_1_ and P_1`_residues of AAS27 are Arg-Ile [26] which corresponds to typical cleavage sites for trypsin-like proteases. This prediction is in accordance with the inhibitory profile described here: rAAS27 is a trypsin and plasmin inhibitor, displaying stoichiometry of inhibition (SI) indices of 3.5 and 3.8, respectively. AAS27 interacts with proteases via a classical “suicide inhibition” mechanism of serpins which involves its cleavage of RCL and formation of S4 β-strand that is inserted in the middle of β-sheet A [51]. We observed the formation of irreversible complexes between rAAS27 with these proteases (Fig 4), which is consistent with typical mechanism of inhibitory serpins. Furthermore, AAS27 inhibited trypsin with *k*_*a*_ of 6.46 ± 1.24 x 10^4^ M^-1^ s^-1^, which is comparable to other tick serpins. A *k*_*a*_ of 9.3 × 10^4^ M^−1^ s^−1^ was described for the cattle tick serpin RmS-15 and thrombin [52]. Prevot et al., (2006) [53] described second order constants for the *Ixodes ricinus* serpin Iris for neutrophil elastase (4.7 x 10^6^ M^-1^ s^-1^), porcine pancreatic elastase (2.2 x 10^5^ M^-1^ s^-1^), t-PA (2.9 x 10^5^ M^-1^ s^-1^), factor Xa (1.7 x 10^5^ M^-1^ s^-1^), thrombin (2.5 x 10^4^ M^-1^ s^-1^) and trypsin (1.5 x 10^4^ M^-1^ s^-1^). Using higher rAAS27:protease ratios, rAAS27 also inhibits chymotrypsin, factor XIa, and rat trypsin IV (Fig 2), albeit with very high SI indices (Fig 3). The high SI index could be explained by the possibility that AAS27 requires a co-factor to enhance inhibition. It is well documented GAGs act as co-factors in order to accelerate the inhibition rate of serpins [31]. Our findings that AAS27 does not bind two of the four main classes of GAGs suggests this is not the case for this tick serpin.

A typical inhibitory serpin forms a covalent complex with its cognate protease which is resistant to SDS and thermal denaturation, has a stoichiometry of inhibition (SI) close to 1 and a *k*_*a*_ ≥ 10^5^ M^-1^ s^-1^ [50]. Although desired, these features (SI close to 1 and *k*_*a*_ ≥ 10^5^ M^-1^ s^-1^) are not always observed, even for well characterized mammalian endogenous serpins controlling important physiological proteases in mammals. In this very important review about serpins, Gettins (2002) [50] describes several serpins with important physiological functions in mammals with *k*_*a*_ ≤ 10^5^ M^-1^ s^-1^. Similarly, according to the nature of serpin inhibition mechanism, the balance between substrate and inhibition reactions leads to the useful concept of a “stoichiometry of inhibition—SI”, which is defined as the ratio of mols of serpin needed to inhibit one mol of protease. However, for a given serpin/protease reaction, there are other factors that can also influence the relative rates of reactive center loop insertion and substrate cleavage and hence affect SI. These include time of incubation, temperature, pH, ionic strength, presence of known and unknown cofactors that differentially affects reaction [50]. We performed all kinetics at 30°C, following recommendations from IUBMB for enzymatic kinetics. We understand that performing the same experiment at 37°C could impact on SI and *k*_*a*_ values observed. In the same way, longer incubation time would impact SI obtained here. For some serpins described in literature with SI close to 1, incubation of serpin and protease were incubated for 2 and even 4 hours [34]. Taken together, we conclude that AAS27 has a physiological relevance for the inhibition of proteases described here, where we demonstrate that rAAS27 is an inhibitor of trypsin and plasmin.

Plasmin inhibition by a tick salivary protein can, at a glance, be viewed as contradictory since plasmin is mostly known for its role in fibrinolysis, an activity that seems beneficial to tick feeding. However, plasmin has also been reported to participate in several processes such as pro-inflammatory cytokine release [38], inducing monocyte and dendritic cell chemotaxis [54], modifying IL-8 and producing a potent attractant of neutrophils [55], tissue remodeling and wound healing [56], all of which can negatively impact tick-feeding success. Thus, inhibition of plasmin by AAS27 seems to contribute to feeding on blood and can be claimed as an important target regarding tick feeding physiology. Similarly, although trypsin is produced predominantly by the pancreas as a means to degrade dietary proteins, trypsin-like proteases are also expressed in the nervous system and in epithelial tissues, where they are the most powerful activators of protease-activated receptor 2 (PAR-2) hence are important factors in neurogenic inflammation and pain in the skin [36]. There is evidence that injection of formalin into mouse paw release locally several forms of active trypsin-like serine proteases. These proteases generate PAR-derived peptides and activates cells via PAR-2-dependent mechanism, resulting in an acute inflammatory response characterized by edema formation in the paw [36,37].

Formalin induces a transient paw edema formation in rats or mice through trypsin IV-induced PAR-2 activation and is inhibited by administration of serine protease inhibitors [36,37]. Although rAAS27 is not an efficient inhibitor of rat trypsin IV, we demonstrate that it inhibits formalin-induced acute inflammation in two different models, the paw edema and vascular permeability assays (Fig 9 and Fig 10). Thus, tick injection of AAS27 into the feeding site could interfere with serine protease-derived pro-inflammatory and algesic responses in the skin during tick feeding.

The observation that a monospecific antibody against rAAS27 abolished its inhibitory activity (Fig 8) is significant because it suggests the potential to design anti-tick vaccines that can neutralize functions of tick serpins at the feeding site. Host functional antibodies raised during immunization could block serpin inhibitory activity during tick feeding and interfere with modulatory functions of serpins, impairing blood meal acquisition, tick development, and pathogen transmission [57]. Inducing an efficacious immune response against tick serpins could help hosts to limit and control parasite infections and, therefore, serpins may be included as suitable antigen candidates in an anti-tick vaccine.

In summary, this study reports that *A*. *americanum* ticks secrete an anti-inflammatory serpin into host during feeding. We would like to caution the reader about limitations of data reported here: the amount of native AAS27 that *A*. *americanum* ticks inject into the host is unknown. Interestingly, we have recently shown that AAS27 is among the top proteins that are secreted into tick saliva when *A*. *americanum* ticks are stimulated to feed on rabbits or humans or dogs (27), which demonstrates that this protein is among those that regulate early tick feeding events. This work emphasizes the importance of understanding the functional roles of tick saliva proteins as a means to identify new targets for the development of novel strategies to control ticks and tick-borne diseases. Work to validate the potential of AAS27 as a target antigen for anti-tick vaccine development is currently being pursued.

## Materials and methods

### Ethics statement

All animal work was conducted and approved according to Texas A&M University Institutional Animal Care and Use Committee (AUP 2011–0207) and by the Ethical Committee on Research Animal Care of the Universidade Federal do Rio Grande do Sul, Brazil (register number 28371/2015). All the procedures involving animals were carried out in accordance with the U.S. Government Principles for the Utilization and Care of Vertebrate Animals Used in Testing, Research and Training; and by the Brazilian Guide for the Care and Use of Animals for Scientific and Didactic Purposes (DBCA-CONCEA).

### Recombinant protein expression and purification of rAAS27

AAS27-encoding mature protein open reading frame [26] was cloned in frame with α-factor into pPICZαC in *Cla*I and *Sac*II sites using forward (5’- TTTTTTT**ATCGAT**GCTGACAGAGAAGGAGCAGAAGCTCATC-3’) and reverse (5’AAAAAA**CCGCGG**TCAGTGGTGGTGGTGGTGGTGGAGCTTGTTGACCTGTCCAGCAAAG-3’) primers with added restriction enzymes sites (in bold) and a hexa histidine tag (underlined). Recombinant (r)AAS27 expression was performed as previously described [32,58]. Expression of rAAS27 was confirmed by resolving samples on a 12% SDS-PAGE for western blot analysis using anti-C-terminus hexa histidine tag HRP conjugated at 1:5,000 antibody dilution (Life Technologies, Carlsbad, CA, USA) and positive signal was detected using a metal enhanced DAB chromogenic substrate kit (Thermo Scientific, Waltham, MA, USA). The rAAS27 was affinity-purified under native conditions using Hi-Trap Chelating HP Columns (GE Healthcare Bio-Sciences, Pittsburgh, PA, USA). To evaluate purity, affinity-purified rAAS27 was resolved on a 12% SDS-PAGE and stained with Coomassie brilliant blue. Affinity-purified rAAS27 was dialyzed against 20 mM Tris-HCl, NaCl 150 mM buffer pH 7.4, protein concentration determined by BCA (Thermo Scientific, Waltham, MA, USA), and purified protein stored at -80°C upon use.

### N- and O-linked deglycosylation

To determine if rAAS27 was N- and/or O-glycosylated, 5 μg of affinity-purified rAAS27 was treated with deglycosylation enzyme mix according to manufacturer’s instructions (New England Biolabs, Ipswich, MA, USA). Deglycosylation was verified resolving proteins on a 12% SDS-PAGE followed by western blotting analysis using an antibody directed to C-terminus hexa histidine-tag (Life Technologies, Carlsbad, CA, USA) at 1:5,000 dilution and positive signal detected using HRP chromogenic substrate (Thermo Scientific, Waltham, MA, USA).

### Antibody production

Anti-serum against rAAS27 was raised in rabbits by subcutaneously inoculating with 100 μg of rAAS27 emulsified in equal volume of TiterMax Gold adjuvant (Sigma, St. Louis, MO, USA). Following the first inoculation, two 50-μg boosters of rAAS27 with adjuvant were applied at 15-day intervals.

Monospecific antibodies were purified using the Sepharose 4B CNBr-activated resin following manufacturer’s instructions (Sigma-Aldrich, St. Louis, MO, USA). First, 2.3 mg of rAAS27 were coupled to 0.3 g of resin. Subsequently, rabbit anti-rAAS27 immune serum dialyzed in phosphate-buffered saline, pH 7.4, (PBS) was added to rAAS27-Sepharose 4B coupled resin with end-over-end rocking at room temperature for 2 hours. The resin was washed with 30 mL of PBS and the bound monospecific anti-rAAS27 was eluted with ten 1 mL aliquots of 100 mM glycine-HCl, pH 2.4, and neutralized with 50 μL of 2 M Tris base. Eluted antibody fractions were dialyzed against PBS (pH 7.4) and were analyzed by SDS-PAGE for Coomassie blue staining and immunoblot analysis.

### Sequence analysis and comparative modelling

The three-dimensional (3D) structure of AAS27 was predicted using a comparative modeling approach. The native human neuroserpin structure (3F5N) [59] was retrieved from the Protein Data Bank (PDB) (http://www.rcsb.org) and used as a molecular template for AAS27 modeling based on 33% and 56% sequence identity and similarity, respectively. Sequence alignments were generated using the ClustalW algorithm [60] and used as input in the Modeller 9v19 program [61]. Models generated were evaluated using QMEAN4 and PROCHECK to estimate model reliability and predict quality [62,63].

The electrostatic potential of AAS27 model was calculated using the Adaptive Poisson–Boltzmann Solver (APBS), while protonation states were assigned using the parameters for solvation energy (PARSE) force field for each structure by PDB2PQR [64]. Execution of APBS and visualization of resulting electrostatic potentials were performed by using the Visual Molecular Dynamics (VMD) program [65] at ±5 kT/e of positive and negative contour fields.

### Validation of AAS27 glycosaminoglycan-binding sites

Two strategies were used to determine if putative glycosaminoglycan (GAG)-binding sites presents in AAS27 were functional. Firstly, to check if rAAS27 was able to bind heparin, 200 μg of affinity-purified rAAS27 was bound and eluted on a 1 mL HiTrap Heparin HP Column following manufacturer’s instructions (GE Healthcare Bio-Sciences, Uppsala, Sweden). Column was equilibrated using 10 mM sodium phosphate, pH 7.4 as binding buffer. Elution was performed using a step-wise elution gradient of NaCl (0–2 M). Protein content of each chromatographic fraction was monitored at 280 nm and analyzed by 12% SDS-PAGE following silver staining [66].

In addition to heparin-Sepharose chromatography, a microtiter plate-based assay was performed using non-fractioned heparin, heparan sulfate, dermatan sulfate, and chondroitin sulfate. In order to prepare GAG-coated plate surfaces, 200 μL (per well) of a 25 μg/mL GAG solution in binding buffer (100 mM NaCl, 50 mM Na-acetate, 0.2% Tween, pH 7.2) was added to GAG-binding plate wells (Galen Laboratories Supplies, North Haven, CT, USA). Plates were incubated overnight at room temperature. Next day, plates were washed with binding buffer following blocking with 250 μL 1% bovine serum albumin solution (in PBS, pH 7.4), following using binding buffer. rAAS27 was dissolved in binding buffer in different concentrations (0, 1, 2, 5, 10 and 20 μg/mL) and incubated by two hours at 37°C. Plates were washed with binding buffer following incubation with 200 μL anti-C terminal hexa histidine tag antibody (1:5,000 dilution) in blocking solution for 1 hour at room temperature. Following appropriate washes with binding buffer, wells were incubated with 1-Step Ultra TMB ELISA Substrate (Thermo Scientific, Waltham, MA, USA) for 10 min at room temperature following addition of 2 M sulfuric acid. The OD_450nm_ was determined using the Infinite M200 Pro plate reader (Tecan, Männedorf, Switzerland).

### Tick feeding, dissections, total RNA, cDNA synthesis and tick protein extractions

*A*. *americanum* ticks were purchased from the tick laboratory at Oklahoma State University (Stillwater, OK, USA). Feeding was performed as previously described [33]. Five female ticks were manually detached every 24 h for 5 days (24–120 h). Within the first hour of detachment, tick mouthparts were inspected to remove remnant tissue and washed in diethylpyrocarbonate (DEPC)-treated water to prepare for dissection. Dissected tick organs, salivary glands (SG), midgut (MG), and carcass (CA, the remnants after removal of other organs) were placed in 1 mL of Trizol reagent (Life Technologies, Carlsbad, CA, USA) and RNA was extracted according to the manufacturer’s instructions. Total RNA was re-suspended in DEPC-treated water and quantified spectrophotometrically using the Infinite M200 Pro plate reader (Tecan, Männedorf, Switzerland). Up to 1 μg of total RNA was used to synthesize cDNA using the Verso cDNA Synthesis Kit following the manufacturer’s instructions (Thermo Scientific, Waltham, MA, USA) and stored at -80°C upon use.

To prepare tick protein extracts, five ticks from unfed and manually detached at 24, 72 and 120 h post tick attachment were rinsed in sterile PBS, pH 7.4 and processed for dissections. Tick organs including salivary gland (SG), midgut (MG), synganglion (SYN), Malpighian tubule (MT), ovary (OV) and the remnants labeled as carcass (CA) were isolated and placed into IP lysis buffer with protease inhibitor cocktail (Thermo Scientific, Waltham, MA, USA). Protein extracts were homogenized and stored in −80°C. Total proteins were extracted from all life stages of *A*. *americanum* consisting of eggs 22 days post- oviposition (50 mg), and unfed whole ticks of larvae (n = 100), nymphs (n = 15) and both female and male adults (n = 5 each). Prior to extraction, each life stage was washed in 1% bleach, Milli-Q water, 70% ethanol, and Milli-Q water. Egg proteins were extracted by flash freezing in liquid nitrogen and crushing them using a sterile plastic pestle in extraction buffer (1x PBS, 1mM PMSF, 1mM E-64, 10mM EDTA, pH 7.4). To extract proteins from other life stages, cleaned ticks were placed into a sterile 1.5 mL tube and finely chopped using a sharp pair of shears in extraction buffer. All protein extracts were sonicated on ice at 50% amplitude, centrifuged at 10,000 x g for 10 min at 4°C, and the supernantant was separated and stored in -80°C. The proteins in supernatant fractions were quantified using BCA assay (Thermo Scientific, Waltham, MA, USA).

### Temporal and spatial expression analyses of AAS27 mRNA and protein in different life stages and tick organs

Transcription analysis was done by two-step quantitative RT-PCR (qRT-PCR) using Applied Biosystems 7300 Real Time PCR System (Life Technologies, Carlsbad, CA, USA) as previously described [67]. AAS27 specific qRT-PCR primers (For: 5′- CTGCCTCTGGAGTGGTCGGC-3′ and Rev: 5′-GAAAAGCTCCTGCGTACTA-3′) were used to determine transcript abundance in triplicate pools of cDNA (described above). Cycling conditions were the following: stage one at 50°C for 2 min, stage two at 95°C for 10 min, and stage three contained two steps with 40 cycles of 95°C for 15s and 60°C for 1 min. Reaction volumes in triplicate contained ten-fold diluted cDNAs that was originally synthesized from 1 μg total RNA, 350 nM of forward and reverse AAS27 primers, and 1X SYBR Green Master Mix (Life Technologies, Carlsbad, CA, USA). For internal reference control, a forward (5′-GGCGCCGAGGTGAAGAA-3′) and reverse (5′-CCTTGCCGTCCACCTTGAT-3′) primers targeting of 40S ribosomal protein S4 (RPS4; accession number GAGD01011247.1) which is stably expressed in *Ixodes scapularis* during feeding [68] was used. Relative quantification (RQ) of AAS27 transcript was determined using the delta delta Ct method (2^-ΔΔCt^) [69]. The lowest expressed time point was used as a calibrator for each tissue.

To relate native AAS27 protein to tick development and expression in different tissues during feeding, total protein extracts of eggs (at 22 days post oviposition), unfed larvae, nymph, and adult male and female ticks as wells as dissected tick organs (as described above) were subjected to routine western blotting analyses using the monospecific antibody to rAAS27. Samples were loaded and resolved on a 12% SDS-PAGE and transferred onto a PVDF membrane. Membranes were incubated with a monospecific antibody produced against yeast-expressed rAAS27 (0.30 μg/μL in a 1:500 dilution) for 1 hour at room temperature. Purified total IgG from pre-immune serum (0.66 μg/μL in a 1:250 dilution) was used as control. After washes, membranes were incubated with secondary Clean-Blot IP Detection Reagent HRP conjugated (1:500 dilution) (Thermo Scientific, Waltham, MA, USA). After washes, membranes were incubated with GE Healthcare Amersha ECL Prime Western Blotting Detection Reagent for 5 minutes at room temperature and visualized using ChemiDoc XRS+ imager (Biorad, Hercules, CA, USA).

### Determining if native AAS27 is injected into the host during tick feeding

To determine if native AAS27 is injected into the host during feeding, glycosylated and deglycosylated affinity-purified rAAS27 (500 ng) was subjected to routine western blotting analyses using antibodies to replete-fed *A*. *americanum* tick saliva proteins (1:50 and 1:250 dilution). Antibodies to replete-fed tick saliva proteins used here were produced as previously described [70]. To analyze if rAAS27 cross-react with serum from animals infested with other ticks species, rAAS27 (500 ng both glycosylated as well as deglycosylated protein) were subjected to western blotting analysis using rabbit antibodies generated to replete-fed adult and nymph *I*. *scapularis* (1:50 dilution) tick saliva proteins. Antibodies to tick-saliva proteins from replete-fed *I*. *scapularis* were produced as previously published [71]. Monospecific antibody anti-rAAS27 (0.33 μg/μL, 1:2,000 dilution) and rabbit pre-immune serum (1:50 dilution) were used as controls. After washes, membranes were incubated with secondary Clean-Blot IP Detection Reagent HRP conjugated (1:500 dilution) (Thermo Scientific, Waltham, MA, USA). Membranes were incubated with GE Healthcare Amersham ECL Prime Western Blotting Detection Reagent for 5 minutes at room temperature and visualized using ChemiDoc XRS+ imager (Biorad, Hercules, CA, USA).

### Protease inhibitor (PI) profiling ‬

Inhibitory activity of rAAS27 was tested against a panel of 17 mammalian serine proteases related to host defense pathways against tick feeding. Mammalian proteases (per reaction) tested were: pancreatic bovine α-chymotrypsin (1.4 nM), pancreatic porcine elastase (61.8 nM), human neutrophil proteinase-3 (280 nM), human chymase (21.7 nM), pancreatic bovine trypsin (0.3 nM), pancreatic porcine kallikrein (10 nM) (Sigma-Aldrich, St. Louis, MO, USA), human neutrophil cathepsin G (425.5 nM) (Athens Research & Technology), human plasmin (5.3 nM), human factor XIa (3.7 nM), bovine factor IXa (314.4 nM), human factor XIIa (15 nM), human thrombin (19.2 nM) (Enzyme Research Laboratories), human neutrophil elastase (14.9 nM), human t-PA (23.6 nM), human u-PA (29.6 nM) (Molecular Innovations, Inc., Novi, MI, USA), human factor Xa (10 nM) (New England Biolabs), and rat trypsin IV (20 nM) (Tirloni et al, in preparation).

Substrates were used at 0.20 mM final concentration, including N-succinyl-Ala-Ala-Pro-Phe-pNA for chymase, cathepsin G and chymotrypsin, N-benzoyl-Phe-Val-Arg-pNA for thrombin, trypsin, and trypsin IV, and N-succinyl-Ala-Ala-Ala-pNA for pancreatic elastase (Sigma-Aldrich, St. Louis, MO, USA). The substrate D-Pro-Phe-Arg-pNa was used for factor XIa, factor XIIa, and kallikrein, and substrate Bz-Ile-Glu (γ-OCH_3_)-Gly-Arg-pNA for factor Xa (Aniara Diagnostica, West Chester, OH, USA). Substrate H-D-Val-Leu-Lys-pNA was used for plasmin (Chromogenix, Philadelphia, PA, USA). The substrate CH_3_SO_2_-D-CHG-Gly-Arg-pNA was used for factor IXa, u-PA and t-PA (Molecular Innovations, Inc., Novi, MI, USA). The substrate N-methoxysuccinyl-Ala-Ala-Pro-Val-pNA was used for neutrophil elastase and proteinase-3 (Enzo Life Sciences, Farmingdale, NY, EUA).

Reagents were mixed at room temperature in technical triplicates. One micromolar (1 μM) of rAAS27 was pre-incubated with indicated amounts of the protease for 15 minutes at 37°C in 20 mM Tris-HCl, 150 mM NaCl, BSA 0.1%, pH 7.4 buffer. The corresponding substrate for each protease was added in a 100 μL final reaction volume and substrate hydrolysis was measured at OD_405nm_ every 15s for 15 min at 30°C using the Synergy H1 microplate reader (Biotek, Winooski, Vermont, EUA). The percent enzyme activity inhibition level was determined as previously described [32,58]. Data are presented as mean ± standard deviation of three independent replicate readings.

### Stoichiometry of inhibition (SI)

We determined stoichiometry of inhibition (SI) indices against proteases that were inhibited by more than 80% in the PI profiling assay described above. Different molar ratios (serpin:protease) of rAAS27 were pre-incubated for 1 hour at 37°C with constant concentration of trypsin (0.2 nM), chymotrypsin (1.4 nM), factor XIa (3.7 nM), and plasmin (5.3 nM). The residual protease activity was measured using colorimetric substrates specific for each enzyme (as described above). The data were plotted as the residual activity (V_i_/V_0_) versus the inhibitor to enzyme molar ratio. SI or the molar ration of rAAS27 to protease was determined by fitting data onto the linear regression [34].

### Protease complex formation

At varying molar ratios, affinity purified rAAS27 was incubated with trypsin (0.1 μg) and plasmin (0.2 μg) in 20 mM Tris-HCl, 150 mM, NaCl, pH 7.4 buffer for 1 hour at 37°C. Denaturing sample buffer was added to the reaction mix, and incubated at 95°C for 5 min in thermocycler. Samples were subjected to 12% SDS-PAGE following silver staining [66].

### Determination of the rate of stable complex formation

A discontinuous method was used to determine the rate of inhibition (*k*_*a*_) of trypsin by rAAS27 [34]. The pseudo-first order rate constant with trypsin (1.5 nM) and rAAS27 (2.5–25 nM) was determined by incubation for different periods of time (0–15 min) followed by measurement of residual protease activity. The pseudo-first order constant, *k*_obs_, was determined from the slope of a semi-log plot of the residual protease activity against time. The *k*_obs_ values were then plotted against serpin concentration and the slope of the line of best fit gave an estimate of the second-order rate constant *k*_*a*_.

### Detection of native AAS27 and anti-trypsin activity in tick saliva

Collection of unfed adult females *A*. *americanum* tick saliva was performed as previously described [27]. To validate presence of native AAS27 in tick saliva, monospecific antibody produced against yeast-expressed rAAS27 (1.0 μg/μL in a 1:500 dilution) and a purified total IgG from pre-immune serum (0.50 μg/μL in a 1:250 dilution) were used to screen western blots of pilocarpine-induced tick saliva (1 μg total protein). Secondary Clean-Blot IP Detection Reagent HRP conjugated was used at 1:500 dilution (Thermo Scientific, Waltham, MA, USA). Membranes were developed with GE Healthcare Amersham ECL Prime Western Blotting Detection Reagent for 5 minutes at room temperature and visualized using ChemiDoc XRS+ imager (Biorad, Hercules, CA, USA).

To test trypsin inhibitory activity in crude saliva, trypsin (2 nM) was incubated with of *A*. *americanum* saliva (1 μg) in 20 mM Tris–HCl, 150 mM NaCl, Tween 0.01%, pH 7.4, for 15 min at 37°C. Residual protease activity was measured by the addition of chromogenic substrate N-benzoyl-Phe-Val-Arg-pNA (0.2 mM final concentration) in a 100 μL final reaction volume and substrate hydrolysis was measured at OD_405nm_ every 15s for 15 min at 30°C using the Synergy H1 microplate reader (Biotek, Winooski, Vermont, EUA). Data are presented as mean ± standard deviation of three independent replicate readings.

To analyze if tick saliva native AAS27 was able to form covalent complex with trypsin, tick saliva (1 μg total protein) was incubated with trypsin (0.1 μg) in 20 mM Tris-HCl, 150 mM, NaCl, pH 7.4 buffer for 1 hour at 37°C. Denaturing sample buffer was added to the reaction mix, and incubated at 95°C for 5 min in thermocycler. Samples were subjected to 12% SDS-PAGE following western blot analysis using a monospecific antibody against rAAS27 (0.30 μg/μL in a 1:500 dilution) for 1 hour at room temperature. After washes, membranes were incubated with secondary Clean-Blot IP Detection Reagent HRP conjugated (1:500 dilution) (Thermo Scientific, Waltham, MA, USA) and membranes developed as described above.

### Effect of antibodies to rAAS27 on its (rAAS27) inhibitory activity

Purified rabbit monospecific IgG to rAAS27 was used to check if antibody-binding to serpin could block their protease inhibitory activity. rAAS27 (68 nM) was pre-incubated with monospecific rabbit IgG anti-rAAS27 (varying from 0.125 to 2 μM) or purified rabbit IgG from pre-immune sera (2 μM) for 30 min at 37°C before the addition of trypsin (2 nM), following a new 15 min incubation at 37°C. Substrate N-benzoyl-Phe-Val-Arg-pNA was added to a 100 μL final reaction volume (final concentration 0.2 mM) and substrate hydrolysis was measured at OD_405nm_ every 15 s for 15 min at 30°C using the Synergy H1 microplate reader (Biotek, Winooski, Vermont, EUA). The percent enzyme activity inhibition level was determined as previously described [32,58]. Data are representative of three independent replicate readings.

### Paw edema and vascular permeability assay

Adult male Wistar rats were supplied by the Central Animal Facility (CREAL), Universidade Federal do Rio Grande do Sul, UFRGS, Brazil. They were housed in plastic cages (4 animals per cage) within a temperature controlled room (22–23°C, on a 12 h light/dark cycle) and had free access to water and food. For paw edema assay, formalin- and compound 48/80-induced rat paw edema models were used to investigate the potential anti-inflammatory role of rAAS27. Paw volume was measured using a digital pletismometer (Insight, Ribeirão Preto, SP, Brazil). Before paw volume measurement, the paw was marked at ankle in order to immerge always at the same extent into the pletismometer. Subsequently, 50 μL of formalin (3% in saline) or 50 μL of compound 48/80 (1 μg total in saline) was administered by intraplantar injection in the right paw in the absence or presence of endotoxin-free rAAS27 (25 μg per paw in saline). The left paw was used as control and received the same volume of saline. As a control, each group of rats received the same volume of saline (vehicle) in the presence of serpin only (25 μg per paw). As an index of edema formation, paw volume (in millimeters) was measured at 0, 30, 60, 120, 180 and 240 min (for formalin-induced) and 0, 30, 60 and 120 min (for compound 48/80). The assessment of paw volume was performed always three times by the same operator. The increase in paw volume was calculated by subtracting average volume of the right paw by the average volume of the left paw at each time point.

For vascular permeability assay [45], male Wistar rats (weighting between 250–400 g) were anesthetized with intraperitoneal injection of xylazin (10 mg/kg) and ketamine (75 mg/kg). Under anesthesia, rats were injected intravenously (tail vein) with 700 μL of Evans blue dye (50 mg/kg in saline). After 5 minutes, animals were injected intradermally on the dorsal region (100 μL final volume) with: (i) saline, (ii) formalin 2% in saline, (iii) formalin 2% + 25 μg of rAAS27 in saline, and (iv) 25 μg of rAAS27 in saline. Two spots of each treatment were performed per animal (n = 6; 12 spots per treatment). After 60 minutes, animals were euthanized and an area of skin that included the entire injection sites was carefully removed and photographed. Evans blue dye spots on skin were excised and the dye was extracted incubating skin with 2.5 mL of 50% formamide for 24 hours at 55°C. After centrifugation at 4,000 rpm for 10 minutes, absorbance of the supernatant was measured at 620 nm. Unpaired t-test was used for statics analysis and *p* ≤0.05 was considered statistically significant.

## Supporting information

S1 Fig Native AAS27 is expressed in different life stages and tissues and is highly expressed at adult unfed stage. (A) AAS27 qualitative expression analysis in eggs 22 days after oviposition (E), unfed larvae (L), unfed nymphs (N), unfed adult females (F) and males (M). (C-E) AAS27 qualitative expression analysis in unfed (UN), 24, 72, and 120h fed tick dissected salivary glands (SG), midguts (MG), synganglion (SYN), Malpighian tubules (MT), ovary (OV) and remnants as carcass (CA). Protein extracts were subjected to western blotting analyses using a monospecific antibody (purified) against yeast-expressed rAAS27 (0.30 μg/μL in a 1:500 dilution). rAAS-27 (500 ng) was used as positive control (C+).
(TIF)

S2 Fig rAAS27 is expressed as a glycoprotein in *Pichia pastoris*. (A) Daily expression levels of rAAS27 through five days (1–5). Expression of rAAS27 was confirmed resolving samples on a 12% SDS-PAGE following western blotting analysis using an antibody to the C-terminus hexa histidine tag (1:5,000 dilution). Positive signal was detected using a metal enhanced DAB chromogenic substrate kit. (B) Affinity purification of rAAS27. Recombinant protein was affinity-purified under native conditions using Hi-Trap chelating HP columns. Samples were resolved on a 12% SDS–PAGE following Coomassie brilliant blue staining: (1) total protein loaded onto column, (2) column run through, (3–4) binding buffer washes, (MM) molecular mass ladder, (5) 25 mM imidazole elution fractions, (6) 50 mM imidazole elution fractions, (7) 100 mM imidazole elution fractions, (8) 200 mM imidazole elution fractions, and (9) 300 mM imidazole elution fractions. (C) Protein deglycosylation. Purified recombinant rAAS27 treated with deglycosylation enzyme mix (D+) or without treatment (D-) was resolved on a 12% SDS-PAGE followed western blotting using anti-C-terminus hexa histidine tag antibody (1:5,000 dilution). Positive signal was detected using a metal enhanced DAB chromogenic substrate kit.
(TIF)

S3 Fig AAS27 three-dimensional structure has putative basic patches. The AAS27 model was constructed using the coordinates generated with the Modeler 9v19 program and human neuroserpin (PDB, 3F5N) as template. Calculation of the electrostatic potential surface map was generated using the Adaptive Poisson–Boltzmann Solver (APBS) tool in the Visual Molecular Dynamics (VMD) program at ±5 kT/e of positive and negative contour fields. Electrostatic surface potentials are indicated by blue surface for positively, and red surface for negatively charged regions. Basic patches are indicated and marked by a dashed circle.
(TIF)

S4 Fig Glycosaminoglycans likely do not bind onto AAS27 basic patches. (A) Heparin-Sepharose chromatography. Approximately 200 μg of affinity-purified rAAS27 was bound and eluted from a heparin-Sepharose column as described in Materials and Methods. Fractions were subjected to SDS-PAGE 12% following silver staining. Recombinant protein applied onto column (1), run through (2), molecular mass ladder (MM), binding buffer washes (3 and 4), fractions eluted using a step-wise gradient of 0–2 M NaCl (5–10). (B) Glycosaminoglycan-binding microtiter plate-based assay. This assay was performed to test rAAS27 binding capacity using different GAGs: heparin, heparin sulfate, dermatan sulfate, and chondroitin sulfate. Plates were coated with GAGs overnight at room temperature. Next day, plates were washed and blocked with 1% bovine serum albumin solution. Subsequently, the plate was incubated with rAAS27 in different concentrations (0, 1, 2, 5, 10 and 20 μg/mL) for 2 h at 37°C. After washing, the plate was incubated with the antibody to C-terminal hexa histidine tag (1:5,000) for 1 hour at room temperature. Following appropriate washes, wells were incubated with TMB substrate and reaction was read at OD_450nm_.
(TIF)
