# Supplementary figures and images for: Amblyomma americanum serpin 27 (AAS27) is a tick salivary anti-inflammatory protein secreted into the host during feeding
Source: PLoS Negl Trop Dis. 2019 Aug 26;13(8):e0007660. doi: 10.1371/journal.pntd.0007660 (PMC6730956; doi:10.1371/journal.pntd.0007660)

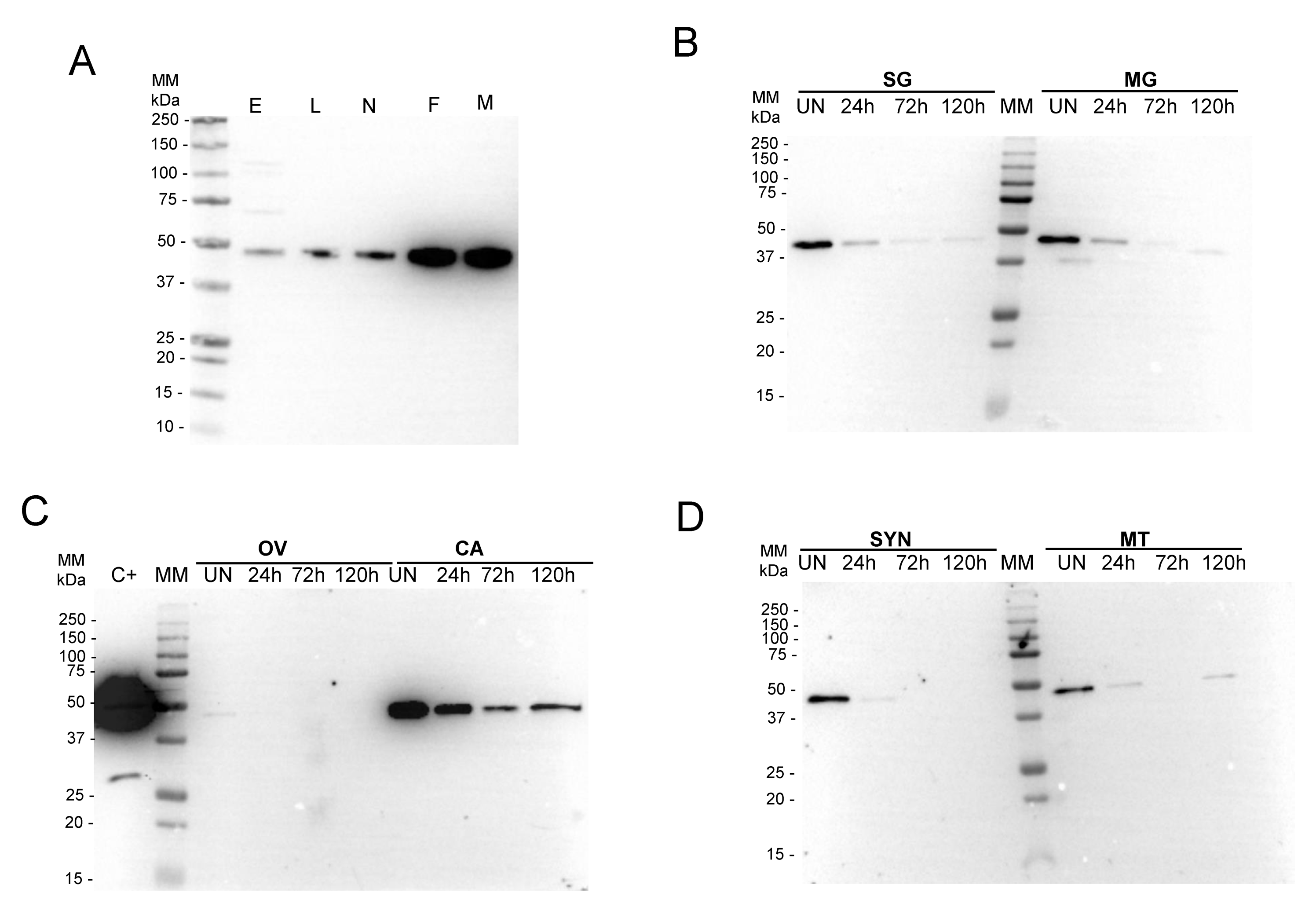

Supplement: S1 Fig — (A) AAS27 qualitative expression analysis in eggs 22 days after oviposition (E), unfed larvae (L), unfed nymphs (N), unfed adult females (F) and males (M). (C-E) AAS27 qualitative expression analysis in unfed (UN), 24, 72, and 120h fed tick dissected salivary glands (SG), midguts (MG), synganglion (SYN), Malpighian tubules (MT), ovary (OV) and remnants as carcass (CA). Protein extracts were subjected to western blotting analyses using a monospecific antibody (purified) against yeast-expressed rAAS27 (0.30 μg/μL in a 1:500 dilution). rAAS-27 (500 ng) was used as positive control (C+). (TIF) [file pntd.0007660.s001.tif]

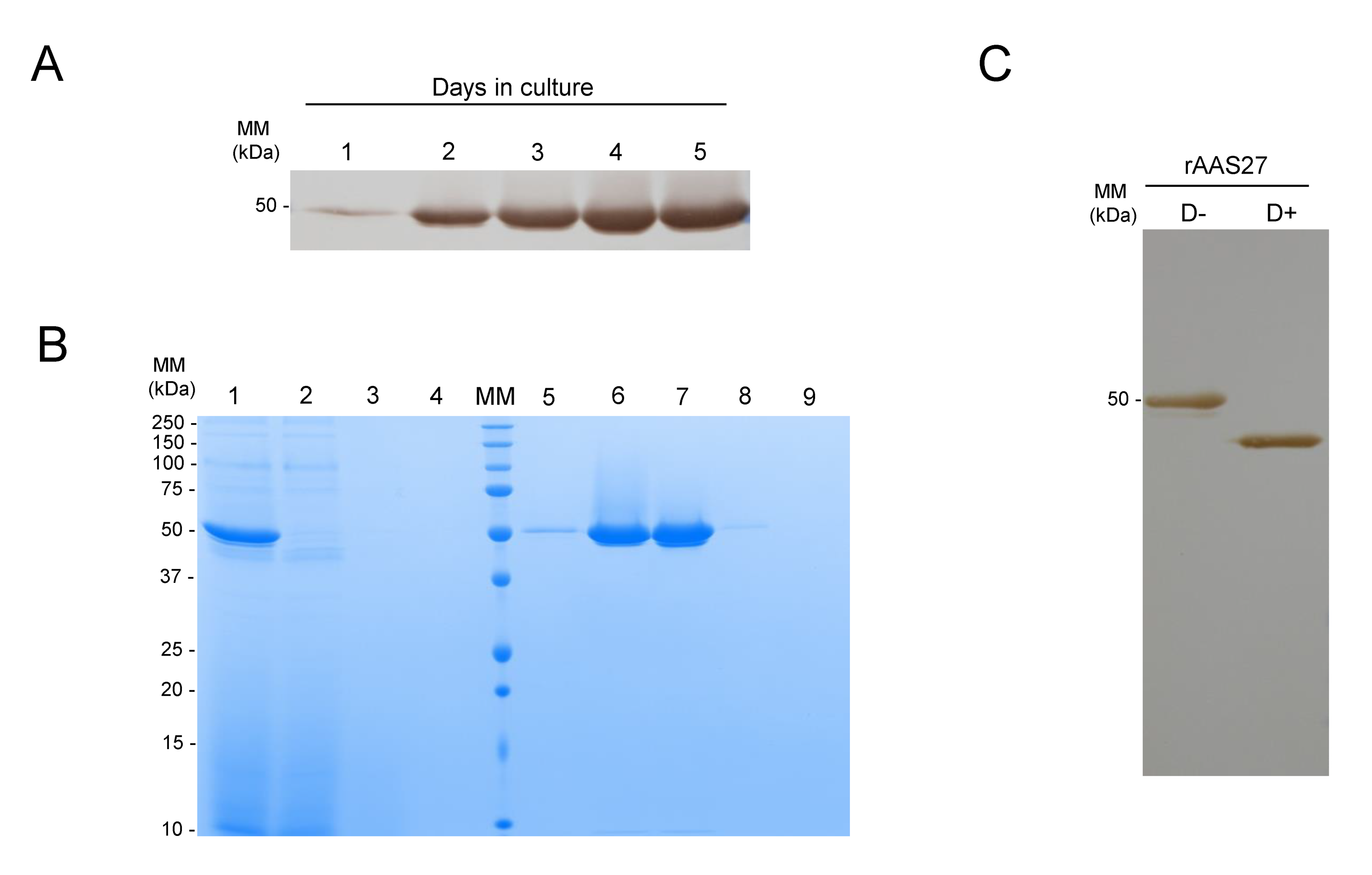

Supplement: S2 Fig — (A) Daily expression levels of rAAS27 through five days (1–5). Expression of rAAS27 was confirmed resolving samples on a 12% SDS-PAGE following western blotting analysis using an antibody to the C-terminus hexa histidine tag (1:5,000 dilution). Positive signal was detected using a metal enhanced DAB chromogenic substrate kit. (B) Affinity purification of rAAS27. Recombinant protein was affinity-purified under native conditions using Hi-Trap chelating HP columns. Samples were resolved on a 12% SDS–PAGE following Coomassie brilliant blue staining: (1) total protein loaded onto column, (2) column run through, (3–4) binding buffer washes, (MM) molecular mass ladder, (5) 25 mM imidazole elution fractions, (6) 50 mM imidazole elution fractions, (7) 100 mM imidazole elution fractions, (8) 200 mM imidazole elution fractions, and (9) 300 mM imidazole elution fractions. (C) Protein deglycosylation. Purified recombinant rAAS27 treated with deglycosylation enzyme mix (D+) or without treatment (D-) was resolved on a 12% SDS-PAGE followed western blotting using anti-C-terminus hexa histidine tag antibody (1:5,000 dilution). Positive signal was detected using a metal enhanced DAB chromogenic substrate kit. (TIF) [file pntd.0007660.s002.tif]

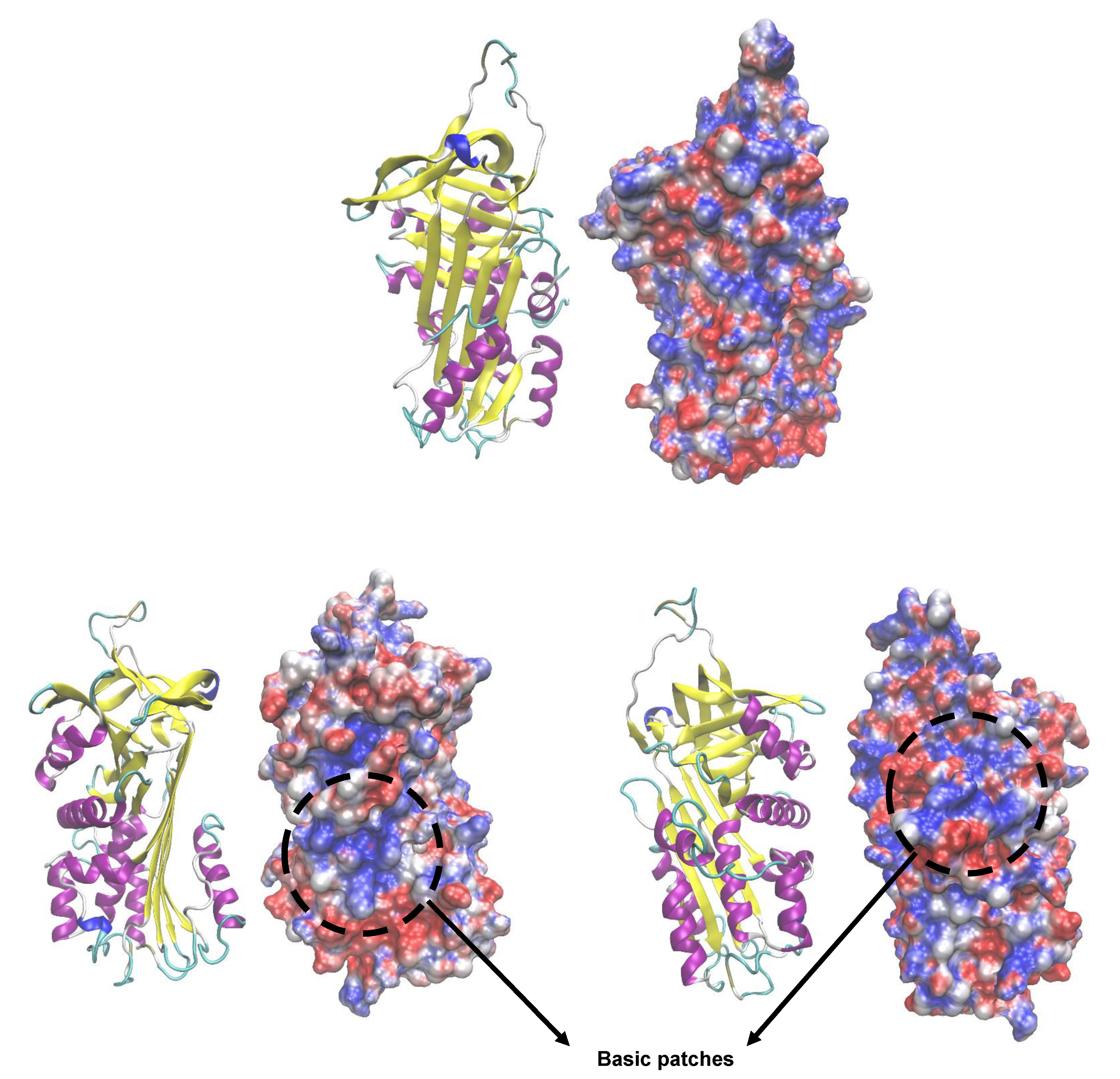

Supplement: S3 Fig — The AAS27 model was constructed using the coordinates generated with the Modeler 9v19 program and human neuroserpin (PDB, 3F5N) as template. Calculation of the electrostatic potential surface map was generated using the Adaptive Poisson–Boltzmann Solver (APBS) tool in the Visual Molecular Dynamics (VMD) program at ±5 kT/e of positive and negative contour fields. Electrostatic surface potentials are indicated by blue surface for positively, and red surface for negatively charged regions. Basic patches are indicated and marked by a dashed circle. (TIF) [file pntd.0007660.s003.tif]

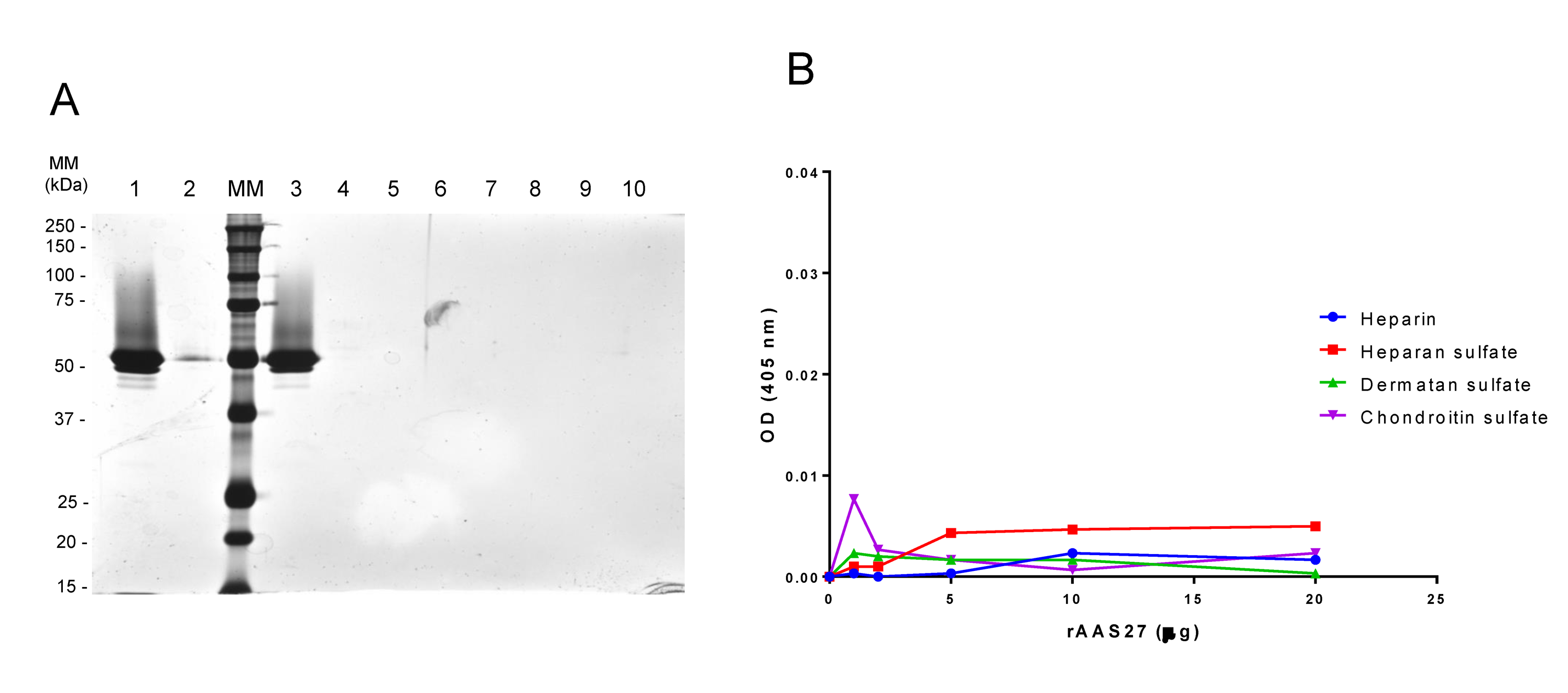

Supplement: S4 Fig — (A) Heparin-Sepharose chromatography. Approximately 200 μg of affinity-purified rAAS27 was bound and eluted from a heparin-Sepharose column as described in Materials and Methods. Fractions were subjected to SDS-PAGE 12% following silver staining. Recombinant protein applied onto column (1), run through (2), molecular mass ladder (MM), binding buffer washes (3 and 4), fractions eluted using a step-wise gradient of 0–2 M NaCl (5–10). (B) Glycosaminoglycan-binding microtiter plate-based assay. This assay was performed to test rAAS27 binding capacity using different GAGs: heparin, heparin sulfate, dermatan sulfate, and chondroitin sulfate. Plates were coated with GAGs overnight at room temperature. Next day, plates were washed and blocked with 1% bovine serum albumin solution. Subsequently, the plate was incubated with rAAS27 in different concentrations (0, 1, 2, 5, 10 and 20 μg/mL) for 2 h at 37°C. After washing, the plate was incubated with the antibody to C-terminal hexa histidine tag (1:5,000) for 1 hour at room temperature. Following appropriate washes, wells were incubated with TMB substrate and reaction was read at OD450nm. (TIF) [file pntd.0007660.s004.tif]
